# Ticagrelor alters the membrane of *Staphylococcus aureus* and enhances the activity of vancomycin and daptomycin without eliciting cross-resistance

**DOI:** 10.1128/mbio.01322-24

**Published:** 2024-09-23

**Authors:** Kirsten Leeten, Nicolas Jacques, Lidia Alejo Esquembre, Dana C. Schneider, Jan Straetener, Camilla Henriksen, Lucia Musumeci, Florence Putters, Sofia Melo, Elena Sánchez-López, Martin Giera, Noémie Penoy, Géraldine Piel, Olivier Verlaine, Ana Amoroso, Bernard Joris, Christoph J. Slavetinsky, Eric Goffin, Bernard Pirotte, Dorte Frees, Heike Brötz-Oesterhelt, Patrizio Lancellotti, Cécile Oury

**Affiliations:** 1 Laboratory of Cardiology, GIGA Research Institute, University of Liège, Liège, Belgium; 2 Department of Microbial Bioactive Compounds, Interfaculty Institute of Microbiology and Infection Medicine Tübingen (IMIT), University of Tübingen, Tübingen, Germany; 3 Department of Veterinary and Animal Sciences, Faculty of Health and Medical sciences, University of Copenhagen, Copenhagen, Denmark; 4 Leiden University Medical Center, Center for Proteomics and Metabolomics, Leiden, the Netherlands; 5 Laboratory of Pharmaceutical Technology and Biopharmacy, Nanomedicine Developments, Center for Interdisciplinary Research on Medicines (CIRM), University of Liège, Liège, Belgium; 6 Bacterial physiology and genetics–Centre d’Ingénierie des Protéines-Integrative Biological Sciences, Department of Life Sciences, University of Liège, Liège, Belgium; 7 Pediatric Surgery and Urology, University Children’s Hospital Tübingen, University of Tübingen, Tübingen, Germany; 8 German Center for Infection Research (DZIF), Partner Site Tübingen, Tübingen, Germany; 9 Cluster of Excellence "Controlling Microbes to Fight Infections (CMFI)", University of Tübingen, Tübingen, Germany; 10 Laboratory of Medicinal Chemistry, Center for Interdisciplinary Research on Medicines (CIRM), University of Liège, CHU Sart Tilman, Liège, Belgium; University of Pretoria, Pretoria, Gauteng, South Africa

**Keywords:** Gram-positive bacteria, drug discovery, drug interactions, platelets

## Abstract

Infections with multidrug-resistant bacteria pose a major healthcare problem which urges the need for novel treatment options. Besides its potent antiplatelet properties, ticagrelor has antibacterial activity against Gram-positive bacteria, including methicillin- and vancomycin-resistant *Staphylococcus aureus* (MRSA and VRSA). Several retrospective studies in cardiovascular patients support an antibacterial effect of this drug which is not related to its antiplatelet activity. We investigated the mechanism of action of ticagrelor in *Staphylococcus aureus* and model *Bacillus subtilis,* and assessed cross-resistance with two conventional anti-MRSA antibiotics, vancomycin and daptomycin. *Bacillus subtilis* bioreporter strains revealed ticagrelor-induced cell envelope-related stress responses. Sub-inhibitory drug concentrations caused membrane depolarization, impaired positioning of both the peripheral membrane protein MinD and the peptidoglycan precursor lipid II, and it affected cell shape. At the MIC, ticagrelor destroyed membrane integrity, indicated by the influx of membrane impermeable dyes, and lipid aggregate formation. Whole-genome sequencing of *in vitro*-generated ticagrelor-resistant MRSA clones revealed mutations in genes encoding ClpP, ClpX, and YjbH. Lipidomic analysis of resistant clones displayed changes in levels of the most abundant lipids of the *Staphylococcus aureus* membrane, for example, cardiolipins, phosphatidylglycerols, and diacylglycerols. Exogeneous cardiolipin, phosphatidylglycerol, or diacylglycerol antagonized the antibacterial properties of ticagrelor. Ticagrelor enhanced MRSA growth inhibition and killing by vancomycin and daptomycin in both exponential and stationary phases. Finally, no cross-resistance was observed between ticagrelor and daptomycin, or vancomycin. Our study demonstrates that ticagrelor targets multiple lipids in the cytoplasmic membrane of Gram-positive bacteria, thereby retaining activity against multidrug-resistant *staphylococci* including daptomycin- and vancomycin-resistant strains.

**IMPORTANCE:**

Infections with multidrug-resistant bacteria pose a major healthcare problem with an urgent need for novel treatment options. The antiplatelet drug ticagrelor possesses antibacterial activity against Gram-positive bacteria including methicillin-resistant and vancomycin-resistant *Staphylococcus aureus* strains. We report a unique, dose-dependent, antibacterial mechanism of action of ticagrelor, which alters the properties and integrity of the bacterial cytoplasmic membrane. Ticagrelor retains activity against multidrug-resistant staphylococci, including isolates carrying the most common *in vivo* selected daptomycin resistance mutations and vancomycin-intermediate *Staphylococcus aureus*. Our data support the use of ticagrelor as adjunct therapy against multidrug-resistant strains. Because of the presence of multiple non-protein targets of this drug within the bacterial membrane, resistance development is expected to be slow. All these findings corroborate the accumulating observational clinical evidence for a beneficial anti-bacterial effect of ticagrelor in cardiovascular patients in need of such treatment.

## INTRODUCTION

Infections with multidrug-resistant (MDR) bacteria are a major healthcare problem with prolonged hospitalization and increased mortality (1). In 2019, around 4.95 million deaths worldwide were reported to be related to antimicrobial resistance of which more than 100,000 were attributed to methicillin-resistant *Staphylococcus aureus* (MRSA) (2). The increasing infection rate with MDR bacteria urges the need for novel treatment options to overcome the problem of untreatable MDR bacterial infections. An emerging avenue of interest involves the repurposing of existing drugs for tackling MDR infections.

One such potential drug that has recently garnered interest is the cyclopentyl-triazolopyrimidine antiplatelet drug ticagrelor. The reversible P2Y12 inhibitor is currently used to prevent cardiovascular events in patients with coronary artery disease (3, 4). Besides its potent antiplatelet properties, a sub-analysis of the PLATO trial comparing ticagrelor and clopidogrel in acute coronary syndrome patients revealed a lower risk of infection-related death upon treatment with ticagrelor (5). This was further supported by the XANTHIPPE study, showing that pneumonia patients experienced enhanced lung function after receiving ticagrelor treatment (6). Following these clinical observations, our team found that ticagrelor has antibacterial activity *in vitro* against Gram-positive and resistant Gram-positive bacteria such as MRSA and methicillin-resistant S*taphylococcus epidermidis* (MRSE) with minimal inhibitory concentrations (MIC) of 20 µg/mL (7, 8). Although 20 µg/mL ticagrelor is above the plasma concentrations reached in conventionally dosed patients, several beneficial effects have been reported at conventional doses that could explain the *in vivo* observations in patients. Our team has shown that ticagrelor at a conventional dose (1 µg/mL) alters S. *aureus* virulence and downregulates essential factors of the accessory gene regulator (agr) system, without affecting bacterial growth. Accordingly, at this dosage, ticagrelor was able to prevent infective endocarditis development in mice while clopidogrel had no effect (9). Moreover, treatment with ticagrelor cured an *S. aureus* infection in a mouse model of prosthetic joint infection, with downregulation of biofilm-related genes (10). These results were further supported by several retrospective clinical studies showing a 1-year lower risk of *S. aureus* bacteremia in acute coronary syndrome patients on ticagrelor compared to clopidogrel (11, 12).

Despite compelling evidence for the antibacterial activity of ticagrelor in addition to its role as a potent antiplatelet drug, its mechanism of action against Gram-positive bacteria remains to be elucidated. The present study aims to identify ticagrelor targets in MRSA and *B. subtilis* and assess its interaction with conventionally used mainstays of anti-staphylococcal therapy, daptomycin and vancomycin. The activity of ticagrelor against daptomycin and vancomycin-resistant strains was also investigated.

## RESULTS

### Ticagrelor induces cell envelope stress responses

**Fig 1:**
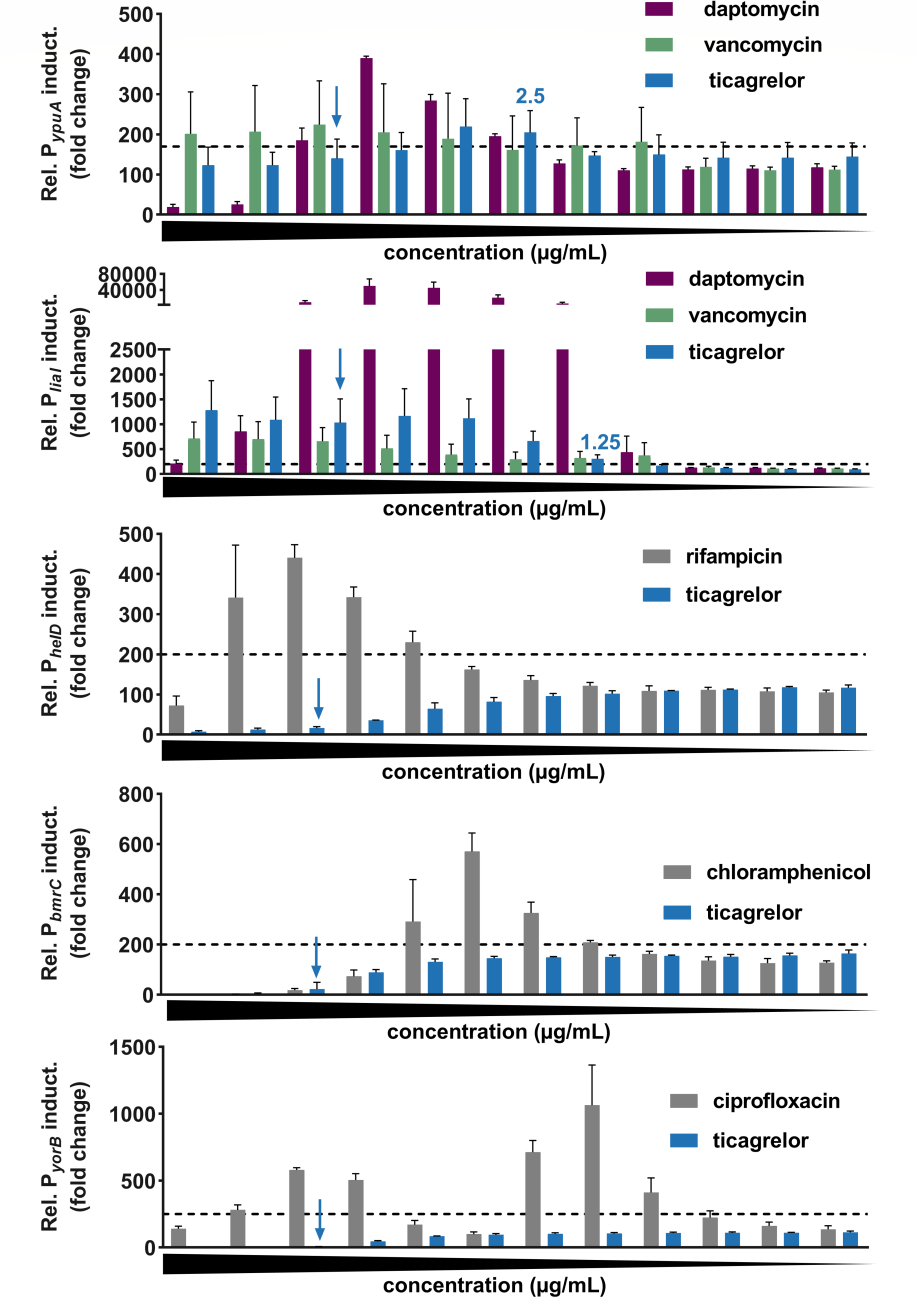
Ticagrelor induces cell envelope-related stress responses in *Bacillus subtilis*. Luciferase-based bioreporter assay using the sporulation-deficient *B. subtilis* 1S34 *luc* reporter strains for *ypuA*, *liaI*, *heID, bmrC*, and *yorB*. Luminescence signals are depicted in % of a control culture, that is, 100% corresponding to the background luminescence level in untreated cells. In a previous study using a large set of reference antibiotics with established modes of action, the threshold values for a significant mode of action-based promotor induction were validated and set to the following promotor-specific threshold values which are indicated by dashed lines (13): 170% for *ypuA* (cell envelope stress) (*N* = 6), 250% for *yorB* (DNA stress) (*N* = 2), and 200% for *liaI* (lipid-II cycle stress) (*N* = 7)*, helD* (RNA stress) (*N* = 3), and *bmrC* (translational arrest) (*N* = 2). Twofold serial dilutions of compounds from left to right. Ticagrelor from 80 µg/mL to 0.08  µg/mL, daptomycin from 32 µg/mL to 0.04 µg/mL, vancomycin from 16 µg/mL to 0.02 µg/mL, rifampicin from 0.006  µg/mL to 0.000003 µg/mL, chloramphenicol from 25 µg/mL to 0.01 µg/mL, and ciprofloxacin from 6.25 µg/mL to 0.003 µg/mL. Graphs represent mean ± SD. The lowest ticagrelor concentration causing induction above the threshold is indicated in blue. Blue arrows mark the MIC concentration of ticagrelor (20 µg/mL).

To narrow down on the cellular structure or metabolic pathway primarily affected by ticagrelor in Gram-positive bacteria, we used a set of five luciferase bioreporter strains in the model species *B. subtilis* which were previously validated with an antibiotic reference library (13). Reporter strains were treated with ticagrelor (0.08 µg/mL to 80 µg/mL) in liquid culture in microtiter plates parallel to reference antibiotics, which were applied in their respective inducing concentration ranges. The cell envelope stress biomarker *ypuA* was induced by 2.5 and 5 µg/mL ticagrelor and a wide range of ticagrelor concentrations (1.25–80 µg/mL) induced the lipid II cycle stress biomarker *liaI* (Fig. 1). In agreement with previous studies (13, 14), these two biomarkers were also triggered by both daptomycin and vancomycin. No induction was observed for the biomarkers of RNA stress (*heID*), translation arrest (*bmrC),* or DNA stress, that is, strand breaks (*yorB*) in contrast to rifampicin, chloramphenicol, and ciprofloxacin, respectively. These results were corroborated in an independent assay format (14) with *B. subtilis* lacZ bioreporter strains (Fig. S1). These data showed that ticagrelor induces cell envelope stress responses, at concentrations below the MIC (20 µg/mL) (Fig. S2A).

### Ticagrelor alters membrane properties of *B. subtilis* and MRSA

**Fig 2:**
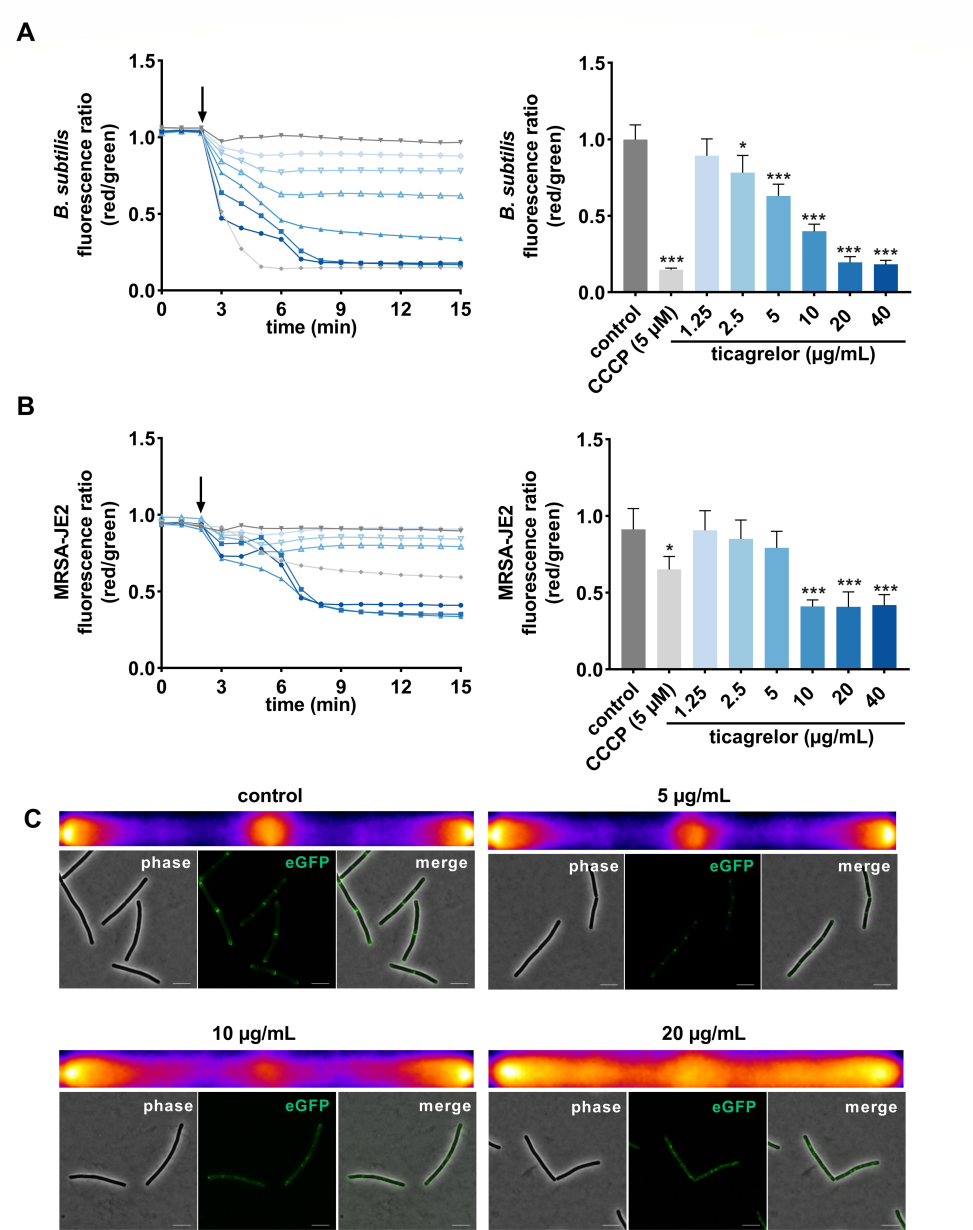
Ticagrelor dissipated the membrane potential of *Bacillus subtilis* and MRSA. Membrane potential over a period of 15 min, based on DiOC_2_(3) staining, expressed as fluorescence ratio (red/green) with the addition (after 2 min, black arrow) of ticagrelor from 1.25 µg/mL to 40 µg/mL for *B. subtilis* 168 (*N* = 3), MRSA USA300 JE2 (*N* = 4), and MSSA (NCTC 8325), with CCCP (5 µM) as a positive control. (**A**) Quantitative analysis of the fluorescence ratio at 6 min after the addition of compounds on *B. subtilis* 168 (*N* = 3), (**B**) *S. aureus* USA300 JE2 (*N* = 4). Graphs show mean ± SD with control (1% DMSO). *P*-values were obtained *via* ANOVA using Dunnett’s multiple comparison test compared to control, with *P < 0.05, and ***P < 0.001. (**C**) Ticagrelor disrupts the septal localization of MinD. High-resolution microscopy images and heat maps of *B. subtilis* GFP-MinD treated with different concentrations of ticagrelor: 5 µg/mL to 20 µg/mL. Heat maps show the average GFP signal for control and ticagrelor-treated cells after 10 min. More than 100 cells were analyzed per condition. The accumulated positional fluorescence information from >100 *B*. *subtilis* cells was transposed into each heat map, the left and right margins represent the cell poles of the rod-shaped cells and the middle corresponds to the septal region. Warmer and brighter colors indicate stronger localization of the fluorescent protein in this position. Scale bar, 5 µm. DiOC2(3), diethyloxacarbocyanine iodide; CCCP, chlorophenyl hydrazone.

We then assessed whether ticagrelor alters the structure or function of the bacterial cytoplasmic membrane using *B. subtilis* and the MRSA strain USA300 JE2. We followed the membrane potential over 15 min using the potential-sensitive fluorescent dye diethyloxacarbocyanine iodide [DiOC_2_(3)]. Ticagrelor triggered a dose dependent and immediate drop of membrane potential in *B. subtilis* (Fig. 2A). Similarly, the membrane potential in MRSA JE2 (Fig. 2B) and MSSA (data not shown) was affected within minutes. For both strains, ticagrelor-induced membrane depolarization reached levels comparable to those obtained with the positive control protonophore, carbonyl cyanide m-chlorophenyl hydrazone (CCCP). Notably, membrane depolarization was induced by sub-inhibitory concentrations of ticagrelor. While the same MIC of 20 µg/mL was obtained for *B. subtilis* and several *S. aureus* strains (MRSA and MSSA) (Fig. S2A through D), effects on the membrane potential were already significant at 2.5 µg/mL for *B. subtilis* and at 10 µg/mL for MRSA USA300 JE2 (Fig. 2A and B). These data were supported by microscopic analyses in a *B. subtilis* strain expressing a GFP-tagged MinD protein (15). This peripheral membrane protein indeed delocalizes upon membrane potential dissipation (15). Delocalization of MinD occurred at 10 µg/mL ticagrelor, and further increased at the MIC (Fig. 2C). As MinD is important for properly positioning the cell division machinery in *B. subtilis* (15), its displacement results in disturbed divisome placement (15). Probing for further topological disturbances, we monitored the position of BODIPY FL-vancomycin, which targets the D-ala D-ala moieties in the peptidoglycan layer and the membrane-bound peptidoglycan precursor lipid II (16). Due to increased peptidoglycan *de novo* synthesis, the septal region contains elevated lipid II levels (17) and is brightly and regularly labeled by BODIPY FL-vancomycin in control cells (Fig. S3A). Microscopy images taken after 1-hour exposure to 10 µg/mL ticagrelor showed aggregates of BODIPY FL-vancomycin distributed within the bacterial cell (Fig. S3A and B), implying lipid II displacement and disturbed peptidoglycan synthesis machinery. Also, the rod-shaped bacteria became shorter and thicker, and cell curvature increased (Fig. S3C through E). Sub-inhibitory concentrations of ticagrelor, therefore, alter both *B. subtilis* and MRSA membrane properties in multiple ways.

### Ticagrelor disrupts the integrity of the cytoplasmic membrane of Gram-positive bacteria

**Fig 3:**
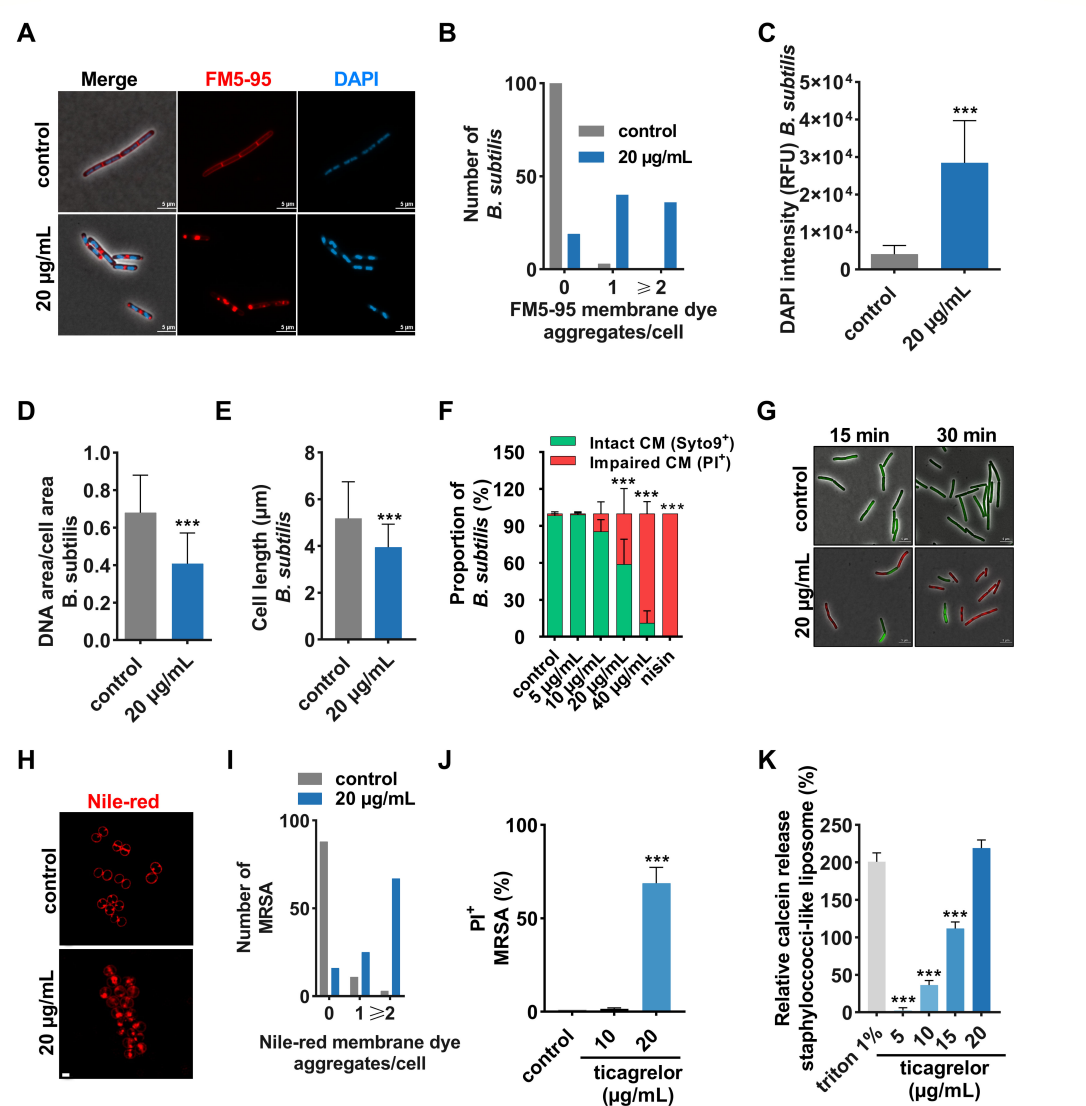
Ticagrelor disrupts the cytoplasmic membrane at the MIC. (**A**) Representative super-resolution microscopy images of *B. subtilis* 168 stained with FM5-95 and DAPI after 15-min treatment with 20 µg/mL ticagrelor (MIC concentration) compared to control, scale bar = 5 µm. Quantification of more than 100 *B. subtilis* cells from three biological replicates: (**B**) FM5-95 aggregates per cell shown as a histogram (Fisher exact test [*P* < 0.001]), (**C**) DAPI permeability, (**D**) DNA condensation, and (**E**) cell length. Graphs show mean ± SD compared to control (1% DMSO). *P*-values were obtained via a *t*-test with ***P < 0.001. (**F**) Quantification and (**G**) microscopy images of PI-positive *B. subtilis* assayed by PI and SYTO9 incorporation after 15 min of treatment with 10 µg/mL to 40 µg/mL ticagrelor compared to 10 µg/mL nisin, more than 100 cells were analyzed. (**H**) Representative super-resolution microscopy images of MRSA BAA-1556 stained with Nile-red after 1-hour treatment with 20 µg/mL ticagrelor compared to control, scale bar = 1 µm. (**I**) Quantification of Nile-red MRSA BAA-1556 membrane aggregates after treatment with 20 µg/mL ticagrelor of more than 100 cells, shown as a histogram (Fisher exact test; *P* < 0.001). (**J**) Quantification of PI-positive MRSA BAA-1556 using FACS after treatment with 10 µg/mL or 20 µg/mL of ticagrelor (*N* = 4). Graphs show mean ± SD with control (1% DMSO). *P*-values were obtained via ANOVA using Dunnett’s multiple comparison test compared to control (1% DMSO), with ***P < 0.001. (**K**) Relative calcein release from calcein-encapsulated unilamellar *Staphylococcus*-like liposomes (60% PG, 25% CL, and 15% LPG) following treatment with ticagrelor from 5 µg/mL to 20 µg/mL, triton 1% was used as positive control. Graphs show mean ± SD with control (1% DMSO). *P*-values were obtained via ANOVA using Dunnett’s multiple comparison test compared to 1% triton, with *P < 0.05, **P < 0.01, and ***P < 0.001. PI, propidium iodide; FACS, fluorescence-activated cell sorting.

At the MIC (20 µg/mL), ticagrelor imposed changes that are characteristics of membrane-active agents in *B. subtilis* (18–22), including the formation of dye aggregates in the membrane (Fig. 3A and B), increased DAPI uptake (Fig. 3A and C), DNA condensation (Fig. 3A and D), and reduced cell length (Fig. 3A and E). The incorporation of the membrane-impermeant dye propidium iodide (PI) into the cytoplasm of *B. subtilis* indicated a breach of the cytoplasmic membrane barrier (Fig. 3F and G). A concentration and time-resolved study (Fig. S4) showed that below the MIC (10 µg/mL), PI could enter only a small fraction of cells (10%–15%). At the MIC, membrane integrity was lost in 40% of cells after 20 min and in almost all cells after 2 h of ticagrelor exposure (Fig. S4A through C). A time-lapse study using the membrane-impermeable dye SYTOX Green as an alternative readout (Fig.S4D) showed membrane disruption by ticagrelor MIC after 10 min, which was almost as rapid as with the pore-former nisin (10 µg/mL) (Movies S1 to S3). The formation of membrane aggregates and PI uptake were also observed at the MIC of ticagrelor in MRSA (BAA-1556) (Fig. 3H through J) and MRSE (Fig. S5). To determine whether ticagrelor can interact with and disrupt lipid membranes without any interaction with a protein component, we performed a calcein release assay using artificial staphylococcus-like phospholipid liposomes (60% phosphatidylglycerol, 25% cardiolipin, and 15% lysophosphatidylglycerol). This assay showed concentration-dependent calcein release, starting at 10 µg/mL, with 20 µg/mL ticagrelor causing complete disintegration of the liposome (Fig. 3K). Altogether, these data indicate that ticagrelor can rapidly kill Gram-positive bacteria, including MRSA and MRSE by disrupting the cytoplasmic membrane.

### Ticagrelor targets multiple lipids in MRSA

**Fig 4:**
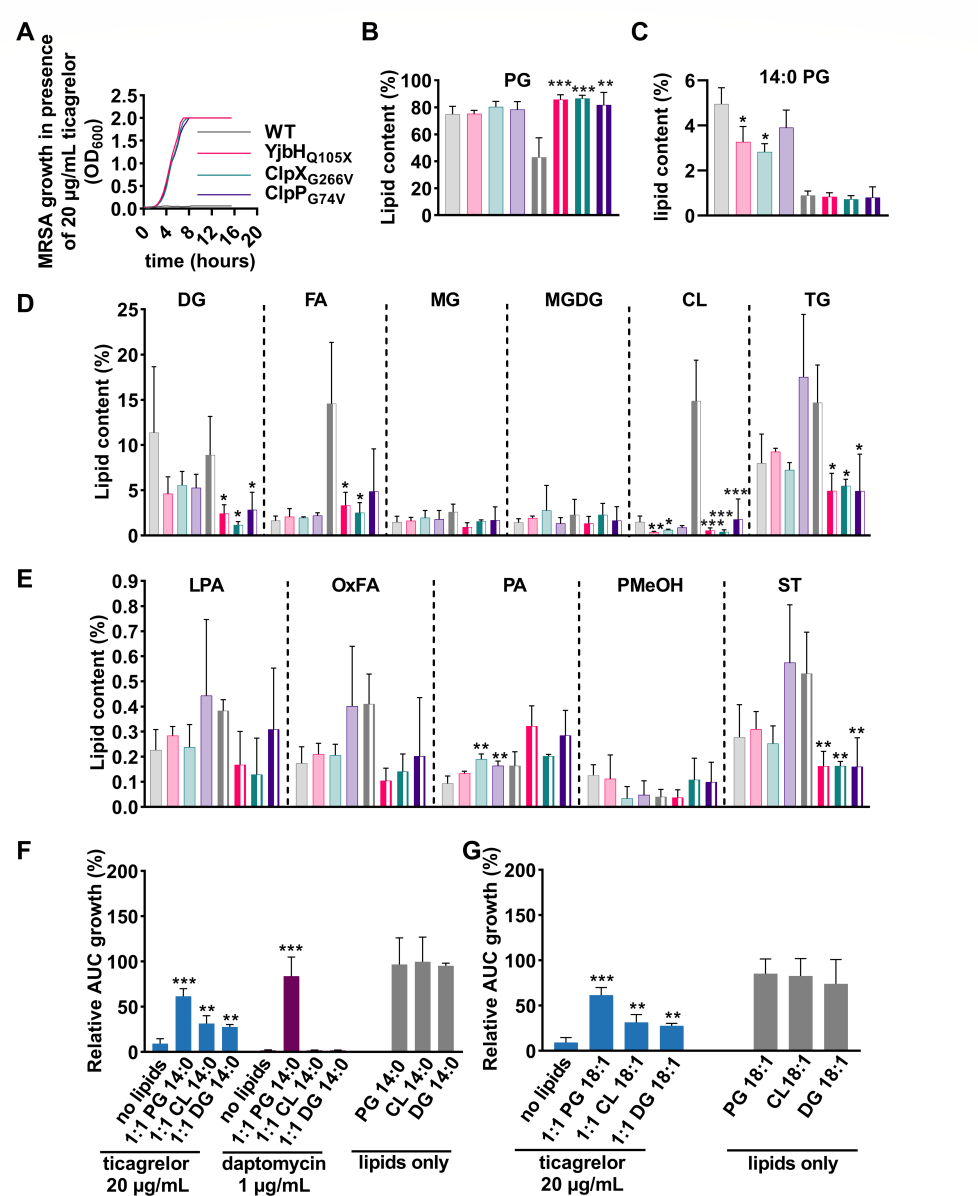
Ticagrelor targets multiple lipids in MRSA. (**A**) Bacterial growth curve of wild-type BAA-1556, YjbH_Q105X_, ClpP_G74V_, or ClpX_G266V_ in the presence of 20 µg/mL ticagrelor (*N* = 3). (**B–E**) Comparative lipidomic analysis of MRSA BAA-1556 WT compared to ticagrelor-resistant clones: YjbH_Q105X_, ClpP_G74V_, and ClpX_G266V_ (*N* = 3). Data are normalized to total lipid content or 14:0 fatty acyl chain-bearing PG, expressed as % lipid content showing exponential (full bar) and stationary phase (striped pattern) bacteria. (**F**) Growth of MRSA BAA-1556 in the presence of 1:1 drug:lipid molar ratio for 20 µg/mL ticagrelor or 1 µg/mL daptomycin and 14:0 fatty acyl chain-bearing PG, CL, or DG with relative AUC growth (%) (*N* = 4). (**G**) Growth of MRSA BAA-1556 in the presence of 1:1 drug:lipid molar ratio for 20 µg/mL ticagrelor and 18:1 fatty acyl chain-bearing PG, CL, or DG with relative AUC growth (%) (*N* = 4). Graphs represent mean ± SD. *P*-values were obtained via ANOVA using Dunnett’s multiple comparison test compared to WT or treatment without lipids with *P < 0.05, **P < 0.01, and ***P < 0.001. AUC, area under the curve; PG, phosphatidylglycerol’ DG, diacylglycerol; FA, fatty acids; MG, monoglycerides; MGDG, monogalactosyldiacylglycerol; CL, cardiolipin; TG, triglycerides; LTA, lysophosphatidic acid; OxFA, oxidized fatty acid; PA, phosphatidic acids; PMeOH, phosphatidyl methanol; ST, sterols; WT, and wild type.

To identify a molecular target of ticagrelor in Gram-positive bacteria, most particularly in MRSA, ticagrelor-resistant MRSA clones were selected *in vitro* using a gradient diffusion method. Nine clones resistant to the highest concentration of ticagrelor (>80 µg/mL) were selected for whole-genome sequencing. Eight clones displayed missense mutations in the *yjbH, clpX,* or *clpP* genes, and one clone had an insertion mutation (position 562) in the yjbK gene (locus tag USA300_FPR3757 SAUSA300_RS04870), encoding for a hypothetical protein (UniProt A0A2S6D7Y3). ClpX and ClpP are the substrate recognition subunits and the proteolytic subunit of the ClpXP protease, respectively, while YjbH is an adaptor protein that targets specific substrate proteins for degradation by bacterial ClpXP (23–25). All mutations in *yjbH* introduced a premature stop codon (amino acids 72, 97, and twice 105), one clone had a mutation in the *clpP* gene (Gly47Val), while the mutations in the *clpX* gene replaced amino acid Gly266 with Val in two clones, and with Cys in one clone. Of note, Gly266 localizes in the IGF motif required for ClpX-ClpP interaction, and substitutions in this motif are known to abolish ClpXP activity in *S. aureus* (24). We selected three different resistance mutations for further analysis, namely the *yjbH* Gln105stop, the *clpX* Gly266Val, and *clpP* Gly74Val. We verified that these mutated MRSA clones were resistant to ticagrelor as shown by normal growth in the presence of ticagrelor MIC (Fig. 4A). Under our *in vitro* growth conditions, growth rates of these three clones were similar to the MRSA parent strain (Fig. S6A). Since no molecular link has been established between the *S. aureus* ClpXP-YjbH protease and the bacterial membrane that we could have directly followed as a lead to the target of ticagrelor, we decided to obtain, as a next step, more insight into the general membrane composition of ticagrelor-resistant *S. aureus*. We performed a high-throughput lipidomic analysis to study changes in lipid composition in ticagrelor-resistant clones and identify possible lipid targets. These experiments made use of both exponential and stationary phase MRSA, characterized by well-known differences in lipid content (26, 27) (Fig. 4B through E; Table S1). Thirteen lipid classes and 97 lipid species were identified in the three mutated clones and the parent MRSA strain. Among the most abundant lipids, these analyses revealed an increased total content of phosphatidylglycerols (PG) in all stationary phase YjbH (Gln105X), ClpP (Gly266Val), and ClpX (Gly74Val) clones compared to parent MRSA, whereas the levels of cardiolipins (CL), diacylglycerols (DG), triglycerides (TG), and free fatty acids (FA) (two clones out of three) were significantly decreased. CL and 14:0 fatty acyl chain-bearing PG levels were also lower in exponential phase Yjbh and ClpX mutants compared to the parent strain (Fig. 4C). In addition, we observed some changes in less abundant lipids, including sterol lipids (ST) that were reduced in all stationary phase mutants, and phosphatidic acid (PA) that was elevated in exponential phase ClpP and ClpX mutants as compared to parent MRSA. These findings indicate major modifications in the lipid composition of ticagrelor-resistant mutants, highlighting potential ticagrelor lipid targets.

Based on this data, a competitive quenching experiment was performed to assess the effect of exogenous lipids, including saturated short fatty acyl chain 14:0 and long 18:0, and mono-unsaturated 18:1 PG, CL, or DG, on the inhibition of bacterial growth by ticagrelor. We first verified that none of the tested lipids affected bacterial growth by themselves (Fig. 4F and G; Fig. S6F through H). All 14:0 and 18:1 lipids tested (PG, CL, and DG) inhibited the antibacterial properties of ticagrelor against MRSA at a 1:1 drug:lipid molar ratio (Fig. 4F and G; Fig. S6B and D), while the 18:0 variants failed to restore growth (Fig. S6E). In contrast to ticagrelor, daptomycin was only antagonized by PG lipids, as shown for 14:0 lipids (Fig. 4F; Fig. S6C). In accordance with these results, external lipids also antagonized the antibacterial activity of ticagrelor on *B. subtilis* (Fig. S7). Growth was fully restored at a drug:lipid molar ratio of 1:1 for CL and 1:2 for PG when 18:1 acyl chain lipids were used. DG had only a minor effect on *B. subtilis*. Similarly, as on MRSA, the 18:0 variants failed to restore growth of *B. subtilis*. These results uncover interactions between ticagrelor and multiple lipids of the bacterial cytoplasmic membrane.

### Absence of cross-resistance between ticagrelor and daptomycin, or vancomycin

**Fig 5:**
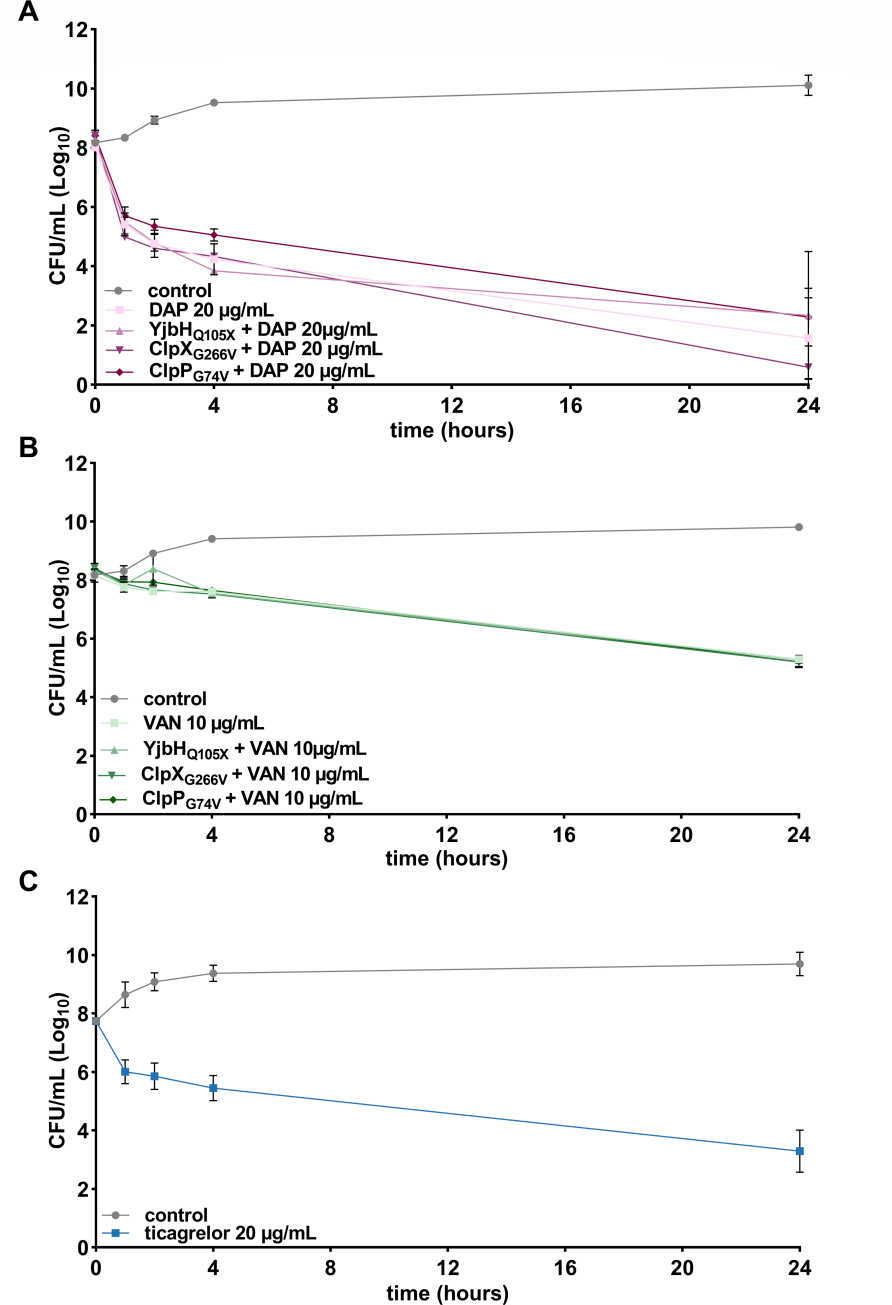
No cross-resistance between daptomycin, vancomycin, and ticagrelor. Time-kill assay of wild-type MRSA BAA-1556 and ticagrelor-resistant clones (YjbH_Q105X_, ClpX_G266V_, and ClpP_G74V_) in the presence of (**A**) 20 µg/mL daptomycin or (**B**) 10 µg/mL vancomycin and (**C**) VISA Mu-50 in the presence of 20 µg/mL ticagrelor. Graphs represent mean ± SD of log_10_ CFU/mL at 0, 1, 2, 4, and 24 hours with control representing 1% DMSO treatment of wild type (*N* = 3). VISA, vancomycin-intermediate *Staphylococcus aureus* ; DAP, daptomycin; VAN, vancomycin.

In view of our findings that ticagrelor has multiple targets in the bacterial membrane, we wanted to assess whether ticagrelor could retain activity against daptomycin-resistant *S. aureus*. A major resistance determinant for daptomycin reported in *S. aureus* is the spontaneous, and commonly observed, acquisition of gain-of-function mutations in the LysPG synthase/flippase MprF. We tested the *in vitro* and *in vivo* selected spontaneous mutations Thr345Ala and Val351Glu in MprF, expressed on a plasmid in the defined background of reference strain *S. aureus* 113 (28), as well as the daptomycin-resistant clinical isolates *S. aureus* 701 and 703, occurring in a patient under daptomycin therapy and both bearing a Ser295Leu MprF variant (29). While the strains showed up to 10-fold elevated MICs for daptomycin, their susceptibility against ticagrelor remained unchanged (Table 1). In addition, an *mprF* deletion mutant of *S. aureus* 113 showed a 13-fold higher susceptibility to daptomycin, while the MIC for ticagrelor was unaffected. In accordance with previous studies, the daptomycin-resistant mutants showed slightly reduced susceptibility to vancomycin (28, 29). Moreover, the ticagrelor MIC against vancomycin-intermediate *Staphylococcus aureus* (VISA) strain Mu50 remained at 20 µg/mL of ticagrelor (Table 1). Furthermore, the ticagrelor-resistant mutants YjbH (Gln105stop), ClpP (Gly74Val), and ClpX (Gly266Val) were equally susceptible to daptomycin and vancomycin, as was the MRSA parent strain BAA-1556. The susceptibility of ticagrelor-resistant mutants to daptomycin and vancomycin was further confirmed in a time-kill assay, which showed identical bactericidal activity of daptomycin (20 µg/mL) and vancomycin (10 µg/mL) on the MRSA parent strain and the ticagrelor-resistant mutants (Fig. 5A and B). In addition, ticagrelor killed VISA and vancomycin-susceptible MRSA with the same efficacy (MBC = 20 µg/mL) (Fig. 5C) (8). Altogether, these data indicate no cross-resistance between ticagrelor and vancomycin or daptomycin.

**TABLE 1:** MIC determination of ticagrelor, daptomycin, or vancomycin against daptomycin (MprF)-, ticagrelor (YjbHQ_105X_, ClpX_G266V_, and ClpP_G74V_)- or vancomycin (VISA Mu50)-resistant strains

		MIC^*a*^ (µg/mL)		
		Ticagrelor	Daptomycin	Vancomycin
*S. aureus* SA113	WT	20 (20/20/20)	2 (2/2/2)	1 (1/1/1)
	∆MprF	26.7 (40/20/20)	0.2 (0.3/0.1/.1)	0.5 (0.5/0.5/.5)
	MprF_T345A_	20 (20/20/20)	9.3 (8/16/4)	1.3 (1/1/2)
	MprF_V351E_	20 (20/20/20)	5.3 (4/8/4)	1.7 (2/1/2)
Clinical isolates *S. aureus*	WT (616)	20 (20/20/20)	0.8 (1/1/.5)	1 (1/1/1)
	MprF_701_	20 (20/20/20)	10.7 (8/16/8)	2.7 (2/2/4)
	MprF_703_	20 (20/20/20)	10.7 (8/16/8)	1.7 (1/2/2)
MRSA BAA-1556	WT	20 (20/20/20)	0.7 (1/0.5/.5)	1 (1/1/1)
	YjbH_G105X_	>80 (>80/>80/>80)	0.8 (1/1/.5)	1 (1/1/1)
	Clp_X266V_	>80 (>80/>80/>80)	0.8 (1/0.5/1)	1 (1/1/1)
	ClpP_G74V_	>80 (>80/>80/>80)	1 (1/1/1)	1 (1/1/1)
VISA Mu50	WT	20 (20/20/20)	2 (2/2/2)	5 (8/4/4)

^*a*^ Mean of three biological replicates, with individual values obtained in three independent repetitions shown in parentheses.

### Ticagrelor enhances the activity of daptomycin and vancomycin

**Fig 6:**
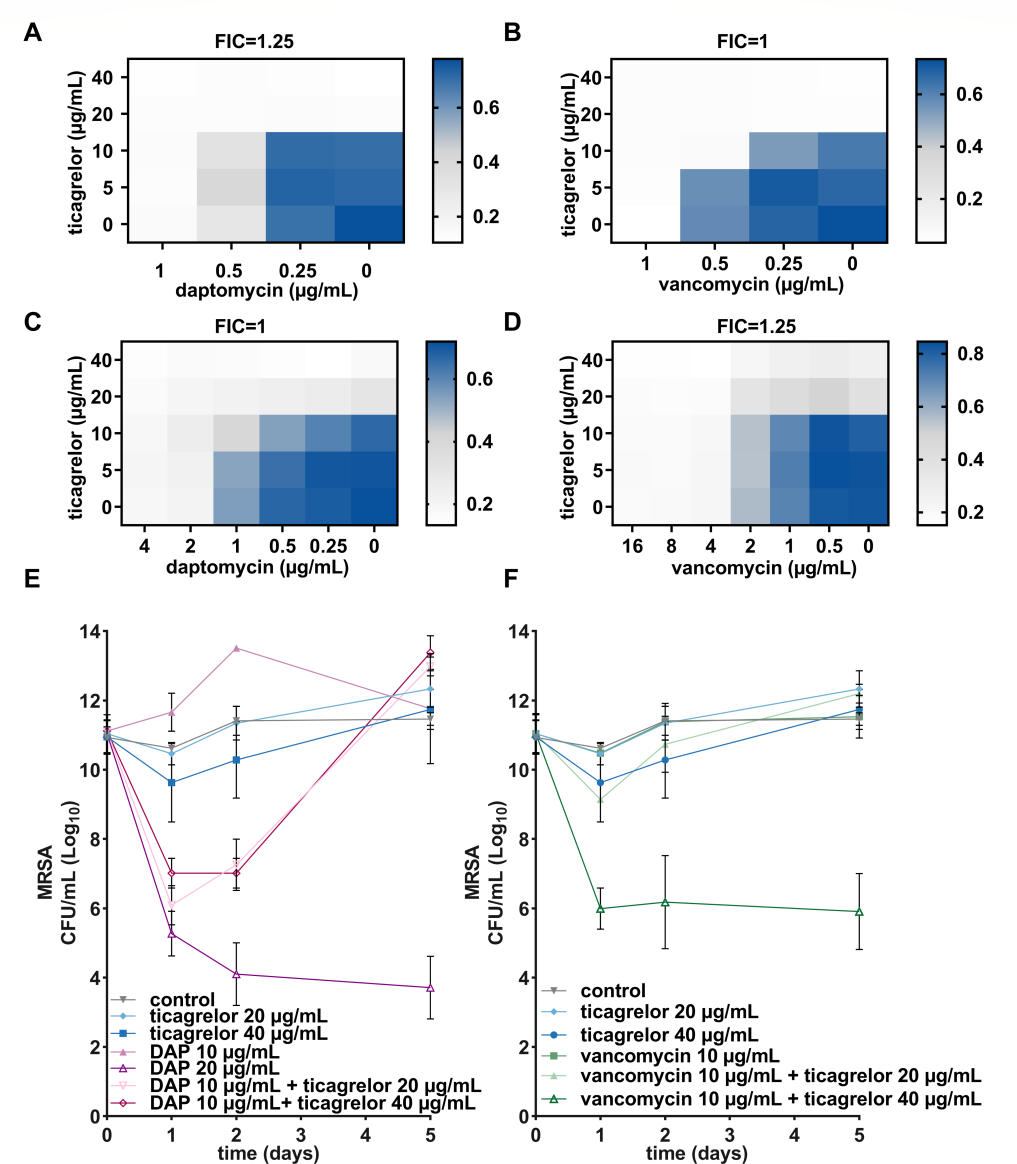
Ticagrelor enhances the activity of daptomycin and vancomycin. Checkerboard assay using MRSA BAA-1556 with a twofold dilution series of (**A**) ticagrelor from 40 µg/mL to 5 µg/mL (vertical) and daptomycin from 1 µg/mL to 0.25 µg/mL (*N* = 5), or (**B**) vancomycin from 1 µg/mL to 0.25 µg/mL (horizontal) (*N* = 3), and (**C**) VISA with a twofold dilution series of ticagrelor from 40 µg/mL to 5 µg/mL (vertical), daptomycin from 4 µg/mL to 0.25 µg/mL (horizontal) (*N* = 6) or (**D**) VISA with a twofold dilution series of ticagrelor from 40 µg/mL to 5 µg/mL (vertical), and vancomycin from 16 µg/mL to 0.5 µg/mL (horizontal) (*N* = 6). Data are represented as a heat map with mean values of ∆OD_600_ determined as the difference in OD_600_ at timepoint 0 and after 24 hours treatment. (**E and F**) Bacterial killing of stationary phase MRSA BAA-1556 by a combination of 20 µg/mL or 40 µg/mL ticagrelor with 10 µg/mL daptomycin or 10 µg/mL vancomycin (*N* = 3). Graphs represent mean ± SD of log_10_ CFU/mL over a period of 5 days with control being 1% DMSO treatment of wild type. VISA, vancomycin-intermediate *Staphylococcus aureus*; DAP, daptomycin; VAN, vancomycin.

We further investigated the interactions between ticagrelor, vancomycin, or daptomycin by combining the drugs two-by-two in checkerboard assays. A combination of a twofold dilution series ranging from 5 µg/mL to 40 µg/mL for ticagrelor and 0.25 µg/mL to 1 µg/mL for daptomycin or vancomycin showed an additive effect against MRSA BAA-1556 with a ΣFIC of 1.25 when combining ticagrelor with daptomycin and a ΣFIC of 1 for vancomycin (Fig. 6A and B). A twofold dilution series from 5 µg/mL to 40 µg/mL for ticagrelor and 0.25 µg/mL to 4 µg/mL for daptomycin also displayed an additive effect on VISA Mu50 with a ΣFIC of 1 (Fig. 6C). A similar additive effect was observed when measuring 5 µg/mL to 40 µg/mL ticagrelor in combination with 0.5 µg/mL to 16 µg/mL vancomycin against VISA Mu50 with a ΣFIC of 1.25 (Fig. 6D). We then assessed the capacity of ticagrelor by its own or in combination with daptomycin or vancomycin to kill stationary phase MRSA. The number of viable bacteria was determined over 5 days. Ticagrelor (up to 40 µg/mL) did not show any bactericidal effect on stationary phase MRSA (Fig. 6E and F). In agreement with previous studies (8, 30), these stationary bacteria were killed by 20 µg/mL daptomycin, which corresponds to its MBC on exponential phase MRSA (Fig. 6E). By contrast, vancomycin was inactive against stationary phase MRSA at the concentration effective against exponentially growing cells (10 µg/mL) (Fig. 6F). Interestingly, combining 40 µg/mL ticagrelor with 10 µg/mL vancomycin could kill stationary phase MRSA (Fig. 6F). In addition, a combination of 20 µg/mL (or 40 µg/mL) ticagrelor with sub-MBC of daptomycin (10 µg/mL) induced a transient decrease in viable bacteria (Fig. 6E). These results are therefore indicative of enhanced antibacterial activity of vancomycin and daptomycin in the presence of ticagrelor.

## DISCUSSION

Here, we show that the antiplatelet drug ticagrelor alters the property and integrity of the cytoplasmic membrane of Gram-positive bacteria in a dose-dependent manner and through a unique mode of action. Notably, this drug retained activity against multidrug-resistant *S. aureus*, including isolates carrying the most common *in vivo* selected daptomycin-resistant mechanism and VISA. We also found that the activity of daptomycin and vancomycin against MRSA was increased in the presence of ticagrelor. Our findings are of clinical importance since these antibiotics are conventionally used to treat MRSA-induced infections and are further relevant considering expanding vancomycin resistance (31).

Similarly to daptomycin and vancomycin, ticagrelor induced bacterial cell envelope stress biomarkers in *B. subtilis* bioreporter strains, pointing to an effect on the bacterial membrane or cell wall (13, 32). Ticagrelor triggered several dose-dependent effects on bacterial membranes that are reminiscent of membrane-active agents (33, 34), including membrane depolarization, the formation of membrane dye aggregates, DNA condensation, and an increased uptake of DAPI and membrane impermeant dyes. Delocalization of the peptidoglycan precursor lipid II and of the cell division regulator MinD, along with changes in cell morphology indicated extensive disturbance of membrane topology, culminating in the loss of membrane integrity allowing dye molecules to enter the cell. Ticagrelor-induced disruption of the bacterial cytoplasmic membrane was demonstrated in several Gram-positive strains, including *B. subtilis*, MRSA, and MRSE. We further confirmed a direct effect of ticagrelor on the integrity of a phospholipid bilayer using staphylococci-like artificial liposomes in a calcein release assay.

The cell wall is the first structure that ticagrelor must pass before reaching the cytoplasmic membrane. In this work, apart from a rearrangement of the lipid II precursor involved in peptidoglycan synthesis by ticagrelor, we did not investigate potential direct interactions of ticagrelor with cell wall components or specific impairment of cell wall biosynthetic reactions. Using thermal proteome profiling, a recent study described that ticagrelor destabilized a subunit of the cell wall teichoic acid translocase in live cells, and two proteins involved in the lipoteichoic acid D-alanylation in cell lysates (35). Impairment of these enzyme activities would increase the net negative charge of *S. aureus* surface, which would result in the observed susceptibility of ticagrelor-treated bacteria to cationic antibiotics such as aminoglycosides and nisin (35). Further investigations might be warranted to characterize the surface polarity and charge of *S. aureus* considering different ticagrelor concentrations, durations of treatment and environmental conditions.

To identify a potential target of ticagrelor, we generated ticagrelor-resistant MRSA clones *in vitro*. Eight of the nine generated clones showed mutations in the *clpP*, *clpX*, or *yjbh* gene. Although no molecular link has been established yet between the ClpXP protease complex and the bacterial membrane, protein degradation through this complex is crucial in controlling stress responses, virulence, and biofilm formation (24, 36–38). *S. aureus* strains with loss of function mutations in ClpXP components are more resistant to certain types of antibiotic stress and show reduced virulence (30, 37, 39). Because these strains are potentially non-infectious, the loss of function mutants have a low occurrence *in vivo*. Interestingly, we previously described that ticagrelor reduces virulence factor expression with a downregulation of several *agr* components (9). While the ClpXP protease has pleiotropic cellular roles, the YjbH adaptor protein specifically targets the transcriptional stress regulator Spx for degradation by the ClpXP protease (23, 36, 40). Inactivation of YjbH prevents Spx from being degraded by the ClpXP protease and is of high relevance, we recently found that high Spx broadly promoted the growth of MRSA in the presence of compounds targeting the cell wall or the cell membrane, including the membrane-specific pore-former nisin (41). Understanding the link between ticagrelor activity or resistance in relation to the ClpXP-YjbH complex will require further investigation, however, increased Spx levels, as a consequence of out-of-function mutations in ClpP, ClpX, or YjbH, are one plausible explanation for the decreased sensitivity of *S. aureus* to ticagrelor.

Preliminary data (not shown) do not indicate that ClpP, ClpX, or YjbH are direct targets of ticagrelor. Instead, the modified lipid composition of generated ticagrelor-resistant clones along with our antibacterial activity antagonizing assays in the presence of exogenous lipids revealed lipid targets for ticagrelor. Among the most abundant lipids in *S. aureus*, we observed lower levels of CL, DG, TG, and FA as well as of 14:0 fatty acyl chain-bearing PG in ticagrelor-resistant clones. Accordingly, our antagonizing assays revealed that PG, CL, and DG restored bacterial growth. Interestingly, we noted a strong influence of the fatty acid side chains, with shorter (14:0) or unsaturated, long (18:1) fatty acids rescuing bacterial growth more strongly than saturated, long acyl chains (18:0), which may imply that lipid fluidity is required for the protective effect. Our data rule out general interactions of ticagrelor with all amphipathic lipids. However, the exact mechanisms underlying ticagrelor-lipid interactions remain to be determined.

In contrast to ticagrelor which showed interactions with multiple lipids, our antagonizing assays indicated that daptomycin is preferentially bound to PG, as compared to CL and DG. Daptomycin is a Ca^2+^-dependent cyclic lipopeptide that is highly potent against MRSA. According to the current model, after binding calcium, Ca^2+^-daptomycin forms at tripartite complex with PG and undecaprenyl-coupled peptidoglycan precursors in *S. aureus* membranes. While, initially, daptomycin binding occurs primarily at the septum affecting peptidoglycan synthesis, major membrane rearrangements follow, resulting in lipid clustering, detachment of peripheral membrane, and gradual membrane disintegration (42–46). Importantly, ticagrelor remained active against daptomycin resistance mutations in MprF, which are commonly selected *in vivo* (28, 29, 47, 48). Recent advances showed that most point mutations in MprF causing daptomycin resistance cumulate in a hot spot region of the MprF flippase part (e.g., T345A, V351E) (28). Since intramolecular domain interactions in the MprF flippase are weakened in these mutants, the authors suggested a mechanistic model in which daptomycin can directly be bound and expelled from the cell by MprF (28). This mechanism is strongly supported by the recently presented cryo-electron microscopy structure of MprF and a computational docking study suggesting that daptomycin can fit well in the active flippase region when above-mentioned point mutations are introduced (49). Therefore, most *S. aureus* resistance mechanisms to daptomycin appear to be tailored to its structure and, in agreement with our data, do not affect ticagrelor sensitivity. *Vice versa*, ticagrelor-resistant strains bearing YjbH_Q105X_, ClpP_G74V_, and ClpX_G266V_ mutations remained sensitive to daptomycin. Besides daptomycin, vancomycin is an antibiotic that is broadly and conventionally used to treat MRSA infections (50). Here, the well-characterized VISA Mu-50 strain was sensitive to ticagrelor, and vancomycin remained active against all ticagrelor-resistant MRSA clones. Our data therefore support the ability of ticagrelor to circumvent these traditional antibiotic resistance mechanisms, thereby offering a potential alternative treatment option for difficult-to-treat infections. In addition, our data indicated that ticagrelor increased the antibacterial activity of daptomycin and vancomycin against MRSA and VISA. When combined with vancomycin, ticagrelor could even kill MRSA in the stationary phase, while the drugs had no effect on their own. Despite additive potential, standard checkerboard assays showed no synergistic effects between ticagrelor and daptomycin or vancomycin, further supporting distinct mechanisms of action. In line with our observations, an additive effect of ticagrelor together with vancomycin was previously reported against *C. difficile* (7).

In conclusion, our study shows that ticagrelor targets major lipids in the Gram-positive bacteria membrane and causes dose-dependent membrane alterations and disruption while retaining activity against multidrug-resistant staphylococci including daptomycin- and vancomycin-resistant strains.

## MATERIALS AND METHODS

Additional materials and methods are described in the supplemental material.

### Bacterial strains and growth conditions

The bacterial strains used in this study are listed in Table S2. Bacteria were aerobically cultured at 37°C under continuous shaking (200 rpm) in indicated media.

### Bioreporter assays

The *B. subtilis 1S34 luc* bioreporter strains using the firefly luciferase as readout were generated and validated by Urban and colleagues (13). The method was adjusted as follows: Overnight cultures of *B. subtilis* P*_ypuA_*, P*_liaI_,* P*_yorB_,* P*_heID_*, and P*_bmrC_ -luc* were diluted to an OD_600_ of 0.05 in lysogeny broth (LB) medium and incubated at 37°C at 190 rpm until an OD_600_ of 0.9 was reached. Bacteria were then diluted in LB medium (P*_ypuA_*, P*_liaI_,* P*_yorB_,* and P*_heID_*) or Belitzky (51) minimal medium (P*_bmrC_*) to an OD_600_ of 0.02 and further diluted 1:1 to medium containing a twofold serial dilution from 80 µg/mL to 0.08 µg/mL for ticagrelor, 12.5 µg/mL to 0.01 µg/mL for vancomycin, 6.25 µg/mL to 0.003 µg/mL for ciprofloxacin, 0.006  µg/mL to 0.000003 µg/mL for rifampicin, 25 µg/mL to 0.01 µg/mL for chloramphenicol, or 32 μg/mL to 0.04 μg/mL for daptomycin and incubated at 37°C for 1 hour in a white flat-bottom 96-well plate. Luminescence was measured in a Tecan SPARK reader 5 seconds after adding 0.1 M citrate buffer (pH 5) containing 2 mM luciferin. The agar-based bioreporter assay method is described in the supplemental material.

### Membrane potential assay

Bacterial cultures were grown in LB medium to an OD_600_ of 0.75. Bacteria were pelleted and resuspended to an OD_600_ of 0.5 in phosphate-buffered saline (PBS) and incubated with 30 µM 3,3′-diethyloxacarbocyanine iodide [DiOC2(3)] for 15 minutes in the dark. Cells were transferred to a black 96-well flat-bottom polystyrene microtiter plate and baseline fluorescence was recorded for 2 minutes. Next, a concentration series of ticagrelor was added and fluorescence was measured for a total of 15 minutes using a spectrophotometer (TECAN Spark) with λ_ex_ of 485 nm and λ_em_ of 530 nm and 630 nm. The protonophore CCCP (5 µM) was used as a positive control.

### Microscopic phenotyping

*B. subtilis* 168 trpC2 cultures were grown in LB medium to an OD_600_ of 0.25 and treated with ticagrelor at the indicated concentrations. *B. subtilis* microscopic phenotyping was performed according to previously described methods (see the supplemental material). For MRSA (BAA-1556) and MRSE, overnight bacterial cultures were grown in a TSB medium followed by treatment with ticagrelor. After 1 hour, MRSA was stained for 5 minutes at 37°C with 5 µg/mL Nile-red. After staining, cells were placed in a Gene Frame (ThermoFisher Scientific) on an agarose pad and imaged using a Zeiss Elyra 7 microscope. Propidium iodide (PI) incorporation by MRSA was analyzed after the addition of 1 µg/mL PI. MRSE PI incorporation was determined via flow cytometry, bacteria were stained with 0.1 mg/mL PI for 5 minutes at 37°C, and PI uptake was recorded using a Cytoflex flow cytometer (Beckman Coulter).

### Calcein-loaded liposomes

Calcein solution (60 mM) was prepared in 5 mM HEPES, pH 7.4. Staphylococcus-like liposomes were composed of lysyl-phosphatidylglycerol (LPG) (15%) (Lipoid S100 579000-1170722-10/919), phosphatidylglycerol (PG) (60%) (Avanti, 841188P), and cardiolipin (CL) (25%) (Avanti, 710335P). Lipids were solubilized in ethanol, mixed, and dried in a rotavapor at 30°C for 1 hour. The thin lipidic film was hydrated directly with the calcein solution and the dispersion was extruded three times per filter (three different sizes of filters were sequentially used 0.4 µm, 0.2 µm, and 0.14 µm). The liposome suspension was then washed four times by ultracentrifugation to remove free calcein (35,000 rpm, 4°C, 2 h). Calcein release was measured for 30 min at 37°C in 100 µL of a solution containing 10 mM Hepes pH 7.4 and NaCl 150 mM.

### Minimal inhibitory concentration determination

The MIC of the test compounds was determined by standardized procedures according to the guidelines of EUCAST or CLSI.

### Generation of ticagrelor-resistant mutants and whole-genome sequencing of resistant strains

Ticagrelor-resistant MRSA (BAA-1556) strains were obtained through a gradient diffusion method. The genomic DNA of nine generated ticagrelor-resistant clones was isolated from bacterial pellets using the High Pure kit (Roche, Life Science). Sequencing was carried out using MiSeq v2 kits following the manufacturer’s standard protocols (Illumina Inc, USA). Sequencing was carried out using MiSeq v2 kits following the manufacturer’s standard protocols (Illumina Inc, USA). Comparative genomics analysis was performed on the nine clones and wild-type MRSA. Fastq files of the isolated mutants were mapped against the MRSA reference genome (USA300_FPR3757). All genomes have been mapped using BWA. Prior to these mappings, the raw data from sequencing have been preprocessed using a pipeline based on BBTools (52). The following steps have been made in order: deduplication, adapter trimming, quality recalibration, error correction, and quality trimming with a minimum set of 8. The variant calling has been made using BBTools with a minimum coverage of 5, a minimum average quality of 15, and a minimum fraction of 75%.

### Lipidomic analyses

MRSA wild-type (BAA-1556) and ticagrelor-resistant strains were grown in the TSB medium. Overnight bacterial cultures were diluted 100 times in fresh TSB and allowed to grow in the exponential phase or overnight until the stationary phase. Bacteria were collected by centrifugation at 14,000 × *g* for 5 minutes and washed once in NaCl 0.9%. All values were normalized to the total amount of lipids and expressed as % of lipid content. Lipids were extracted using a modified MTBE protocol according to Matyash et al. (53). Untargeted lipidomic analysis was performed using an LC-MS/MS-based lipid profiling method as previously reported (54), except for the following parameters: ion source gas 1, 2 and curtain gas were 45, 50, and 35 psi, respectively, and an acquisition range of *m/z* 100–1,800 was applied. In addition, a CL-focused analysis was conducted by adjusting the untargeted lipidomics method so that enhanced MS/MS fragmentation could be obtained assisting CL identification. For this, the collision energy and collision energy spread parameters were set to −60 and −5 V, respectively.

### Lipid-based antagonization assays

The effect of 14:0 PG (840445), -cardiolipin (750332), -DG (800814) and 18:1 PG (840521P), -cardiolipin (710335P), -DG (800811C) (Avanti lipids, Sigma-Aldrich) on the inhibition of bacterial growth by ticagrelor or daptomycin was assessed as follows. Overnight MRSA (BAA-1556) cultures were diluted 1,000 times in TSB medium and mixed with lipids at a 1:1 (drug:lipid) molar ratio. Bacterial growth (OD_600_) was measured over time, and the area under the curve (AUC) was determined and expressed as relative AUC (%).

### Time kill assay

Bacteria were grown in an MHB medium and treated with vehicle or test compounds. The number of live bacteria (CFU/mL) was determined over time.

### Checkerboard assay

Checkerboard assays were performed in MHB medium (after the addition of 50 µg/mL Ca^2+^ for daptomycin) according to the standard procedures.

### Data analysis

Statistical analyses were done using GraphPad Prism 9 software. A parametric *t*-test or ANOVA with Dunnett’s multiple comparison was performed. Categorical data were analyzed via a Fisher exact test. Graphs represent mean ± SD with *P* values: *P < 0.05, **P < 0.01, and ***P < 0.001.

## Data Availability

Sequencing data of ticagrelor-resistant MRSA clones are available in BioProject under accession number PRJNA1063881.

## SUPPLEMENTAL MATERIAL

Supplemental material mbio.01322-24-s0001.pdf Supplemental materials and methods, Figures S1 to S7, Table S2, and movie legends.

Table S1 mbio.01322-24-s0002.xlsx Lipidomics data.

Movie S1 mbio.01322-24-s0003.mp4 Untreated *B. subtilis*.

Movie S2 mbio.01322-24-s0004.mp4 *B. subtilis* with ticagrelor.

Movie S3 mbio.01322-24-s0005.mp4 *B. subtilis* with nisin.

ASM does not own the copyrights to Supplemental Material that may be linked to, or accessed through, an article. The authors have granted ASM a non-exclusive, world-wide license to publish the Supplemental Material files. Please contact the corresponding author directly for reuse.
