# Supplementary material for: Ticagrelor alters the membrane of Staphylococcus aureus and enhances the activity of vancomycin and daptomycin without eliciting cross-resistance
Source: mBio. 2024 Sep 23;15(10):e01322-24. doi: 10.1128/mbio.01322-24 (PMC11481878; doi:10.1128/mbio.01322-24)
Supplement: Supplemental material — s and methods, Figures S1 to S7, Table S2, and movie legends. [file mbio.01322-24-s0001.pdf]

SUPPLEMENTAL APPENDIX

Ticagrelor alters the membrane of *Staphylococcus aureus* and enhances the activity of vancomycin and daptomycin without eliciting cross-resistance

Leeten K *et al.*

CONTENT

Supplemental materials and methods

Supplemental figures S1-7

Supplemental tables S1,2

Supplemental movies S1-3

Supplemental materials & methods

Agar-based bioreporter assay

An agar-based bioreporter assay was performed as described earlier (1) using *B. subtilis 1S34 lacZ* strains. A dose of 20 µg ticagrelor was spotted on agar and incubated overnight, β-galactosidase expression was used as a read-out.

B. subtilis microscopic phenotyping

Quantitative image analysis of bacterial dimensions was performed using MicrobeJ (MicrobeJ – An ImageJ plug-in to analyze bacterial cells. https://www.microbej.com/. Retrieved 8 August 2023). *B. subtilis* 168 trpC2 were stained with 20 µg/mL FM5-95, 1 µg/mL DAPI, 1 µg/mL BODIPY FL-vancomycin or a LIVE/DEAD BacLight bacterial viability kit as previously described (2). Stained cells were transferred onto an agarose slide for visualization. To confirm membrane barrier impairment, cells were pre-dyed with 450 nM SYTOX Green and 0.25 µg/mL FM4-64 for 5 minutes before spotting 1 µL of the pre-dyed cells onto a 1.5 % agarose pad containing 25 % LB medium, 20 µg/mL ticagrelor, 0.5 µg/mL FM4-64, and 150 nM SYTOX green. Nisin 10 µg/mL was included as a positive control and fluorescence was recorded every 6 minutes for 1 hour. Microscopic analysis was done using the Nikon Eclipse Ti-E inverted microscopy. Images were acquired with an Orca Flash 4.0 LT camera (Hamamatsu) and further analyzed and processed using NIS Elements Advanced Research (Nikon). Cells out of focus, located on the margins of the field of view or not properly detected by the software were excluded. Following wavelength settings were used: λ_{ex} 515 nm, λ_{em} 640 nm for FM5-95, FM4-64 and PI; λ_{ex} 358 nm, λ_{em} 461 nm for DAPI; λ_{ex} 488 nm, λ_{em} 523 nm for SYTOX green, BODIPY FL-vancomycin, and Syto9. *B. subtilis* GFP-MinD (3) was incubated overnight at 37 °C in LB supplemented with 50 µg/mL spectinomycin. Overnight cultures were then diluted 1:200 in fresh LB medium

containing 0.5% xylose and further grown to an OD_{600} between 0.2-0.3. Exponentially growing cells were then treated with a dilution series of ticagrelor, and fluorescence was recorded after 10 min treatment with λ_{ex} 488 nm and λ_{em} 507 nm.

Microcalorimetry based antagonization assay

The effect of 14:0 -PG (840445), -cardiolipin (750332), -DG (800814) and 18:1 -PG (840521P), -cardiolipin (710335P), -DG (800811C), 18:0 PG (840465), 18:0 CL (710334), and 18:0 DG (800820) (Avanti lipids, Sigma-Aldrich) on the inhibition of bacterial metabolic activity by ticagrelor or daptomycin was analyzed using the calScreener™ (Symcel AB, Sweden) through continuous measuring of heat flow (µW) for 20 hours. The maximum metabolic rate (µW) was determined using the Symcel Calview software.

B. subtilis 1S34 lial luc antagonization assay

B. subtilis 1S34 *lial luc* was used to determine the effect of exogeneous lipids on the activity of ticagrelor. Lipids were added in a 1:1, 1:2 or 1:4 (drug:lipid) molar ratio to 20 µg/mL ticagrelor. Cultures in late exponential phase (OD_{600} of 0.7-0.9) were diluted in fresh medium to OD_{600} = 0.02. The lipid:ticagrelor mixture was prepared in 100 µL medium in flat-bottom polystyrene 96-well plate in duplicates before adding 100 µL of the cell suspension. OD_{600} was recorded in a microplate reader (Tecan Infinite M nano) over 12 h at 37 °C (measurement every 5 min preceded by 30 s of shaking).

Supplemental figures

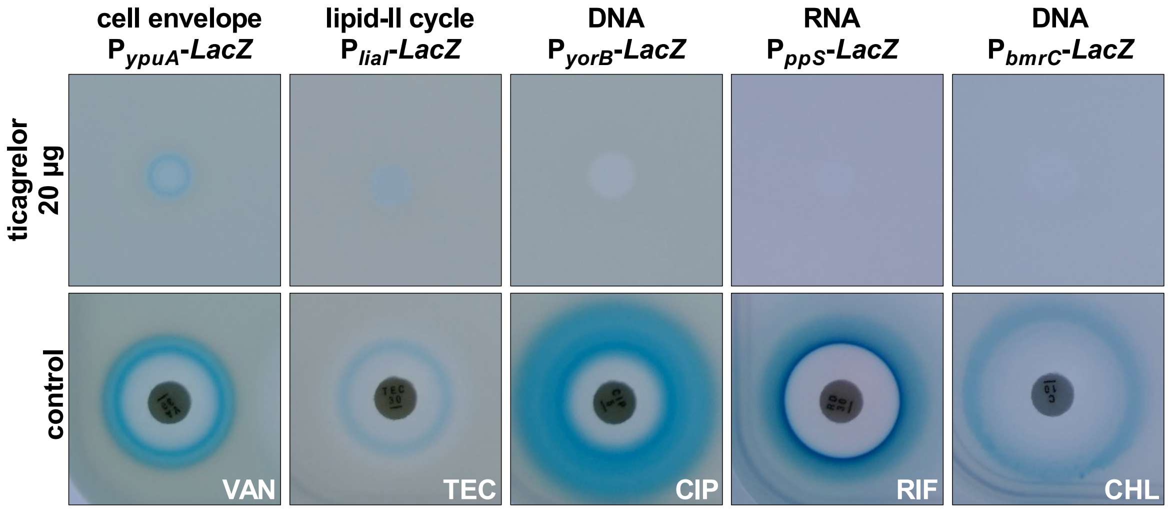

Fig.S1: Agar-based bioreporter screening on *B. subtilis* 1S34 lacZ bioreporter strains using β-galactosidase expression as a readout. Induction of promoters in brackets are indicated by blue halos and signal cell envelope stress (*ypuA*), lipid-II cycle stress (*liaI*), DNA stress (*yorB*), RNA (*ppS*) stress, or translation arrest (*bmrC*). Ticagrelor (20µg) was spotted onto the bioreporter-containing agar and caused a concentration gradient by diffusion. Only the P_{ypuA} and P_{liaI} strains yielded blue halos around the zone of growth inhibition. Reference antibiotics as positive controls: vancomycin 30 µg (VAN), teicoplanin 30 µg (TEC), ciprofloxacin 5 µg (CIP), rifampicin 30 µg (RIF), and chloramphenicol 10 µg (CHL). Pictures are representative of three independent experiments.

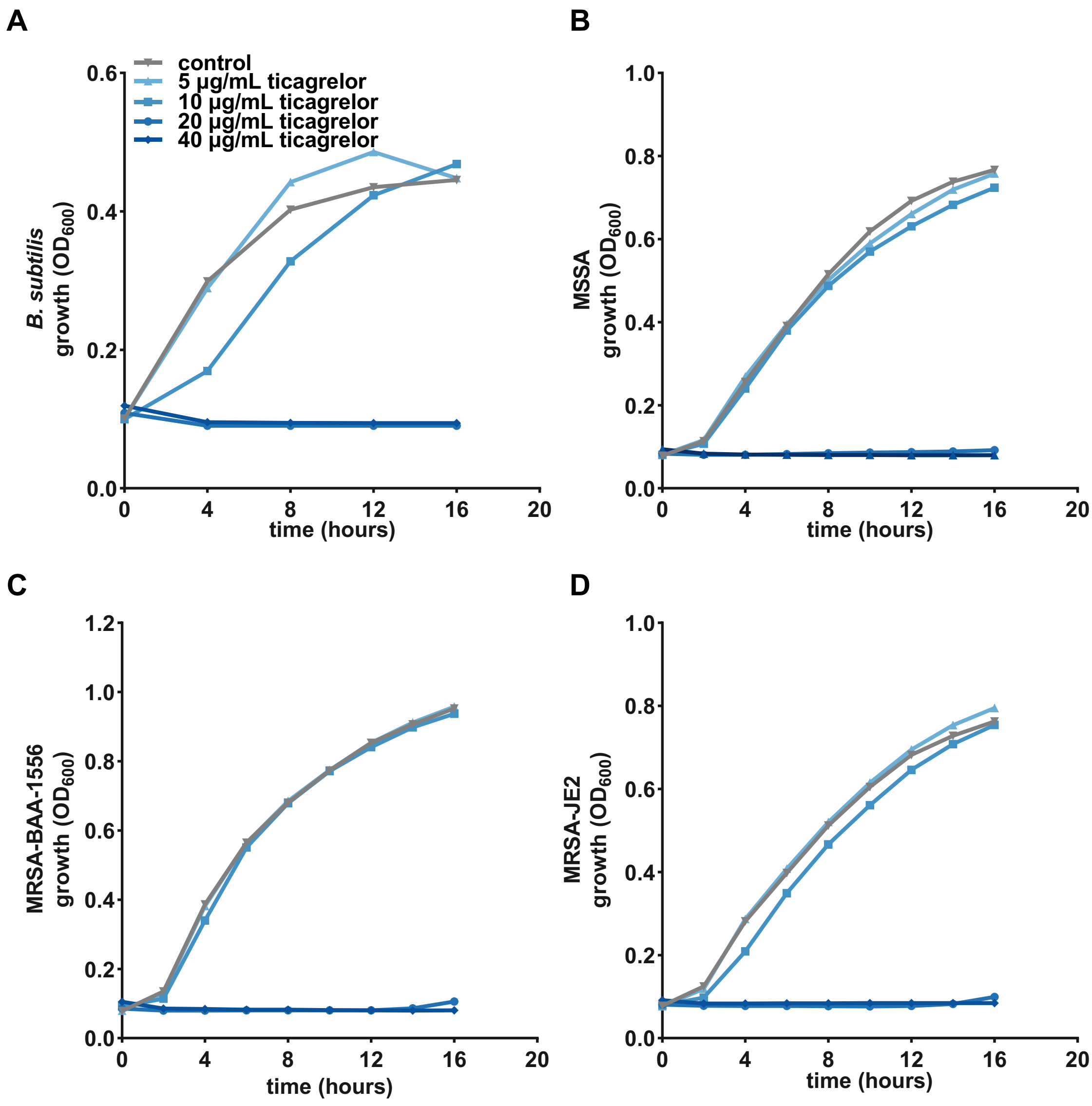

Fig.S2: (A-D) Bacterial growth curve in the presence of 5 μg/mL to 40 μg/mL for *B. subtilis* 168, MSSA (ATCC 6538), and MRSA (BAA-1556, JE2). Graphs show bacterial growth over 16 hours with control (1% DMSO) of three biological replicates.

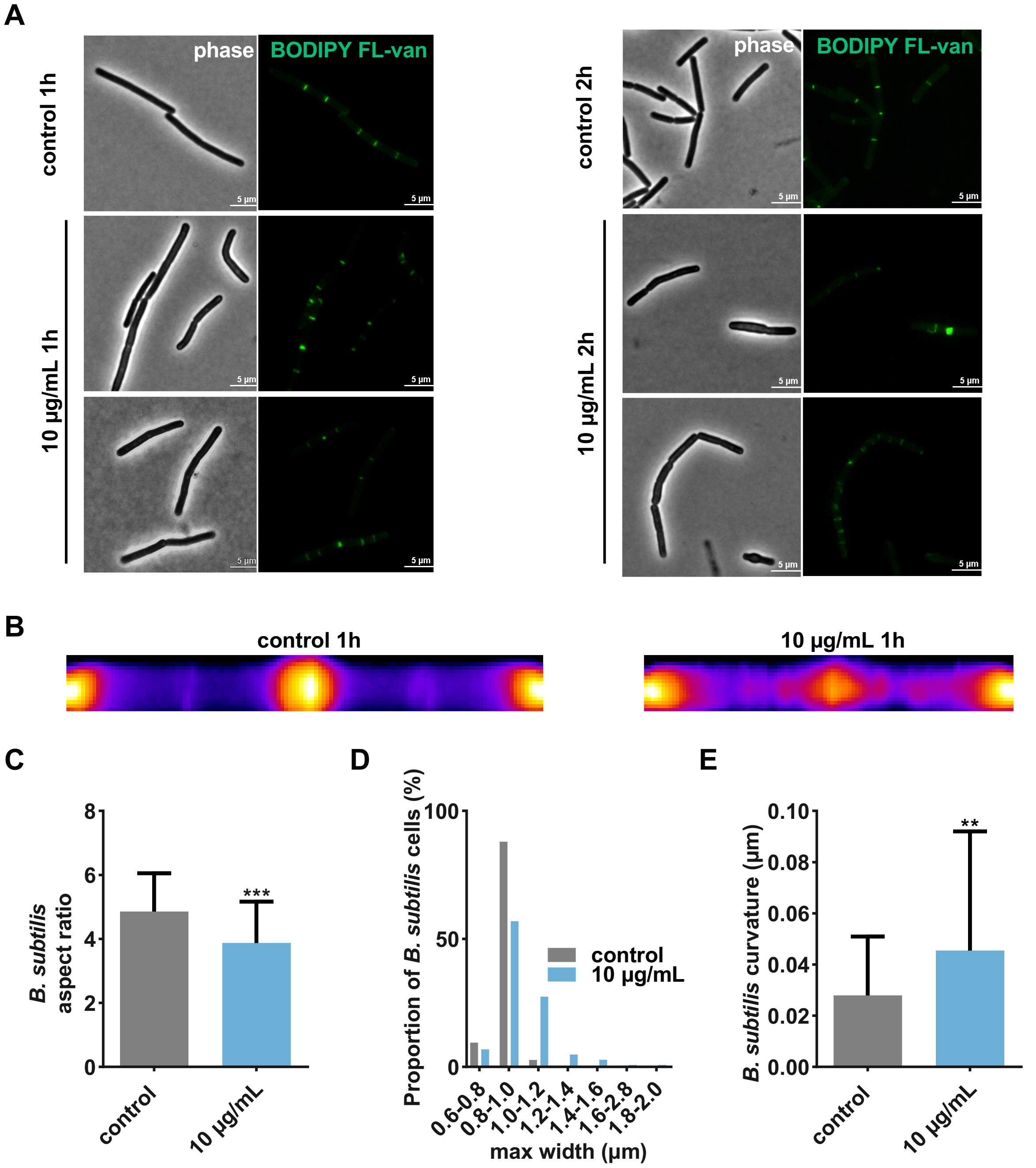
A
phase
BODIPY FL-van
control 1h
10 μg/mL 1h
control 2h
10 μg/mL 2h
5 μm
B
control 1h
10 μg/mL 1h
C
B. subtilis aspect ratio
***
control
10 μg/mL
D
Proportion of B. subtilis cells (%)
control
10 μg/mL
0.6-0.8
0.8-1.0
1.0-1.2
1.2-1.4
1.4-1.6
1.6-2.8
1.8-2.0
max width (μm)
E
B. subtilis curvature (μm)
**
control
10 μg/mL

Fig.S3: Effects of ticagrelor (sub-MIC) on the localization of BODIPY FL-vancomycin and cell shape of *B. subtilis*. (A) 1h and 2h treatment at 10 µg/mL (0.5x MIC), phase contrast and eGFP channel, representative images. (B) Heat maps of the BODIPY FL-vancomycin signal in control cells vs. cells exposed to 10 µg/mL of ticagrelor for 1h; > 100 cells from 3 independent experiments were analyzed and projected into each heat map. (C-E) Quantitative analysis of >100 ticagrelor-treated cells from three independent experiments, 10 µg/mL, 1h treatment. (C) Changes in cell aspect ratio (defined as the ratio of a particle's fitted ellipse, thereby reflecting the length/width proportion). (D) Maximum width (i.e., width at the broadest point of the cell). (E) Sinuosity ratio (defined as the ratio of the curvilinear length and the distance between the end points of the medial axis) as a measure of cell curvature. Graphs show mean ± SD, with control (1% DMSO). P-values were obtained via a t-test with **p-value < 0.01 and ***p-value < 0. 001.Scale bar 5 µm.

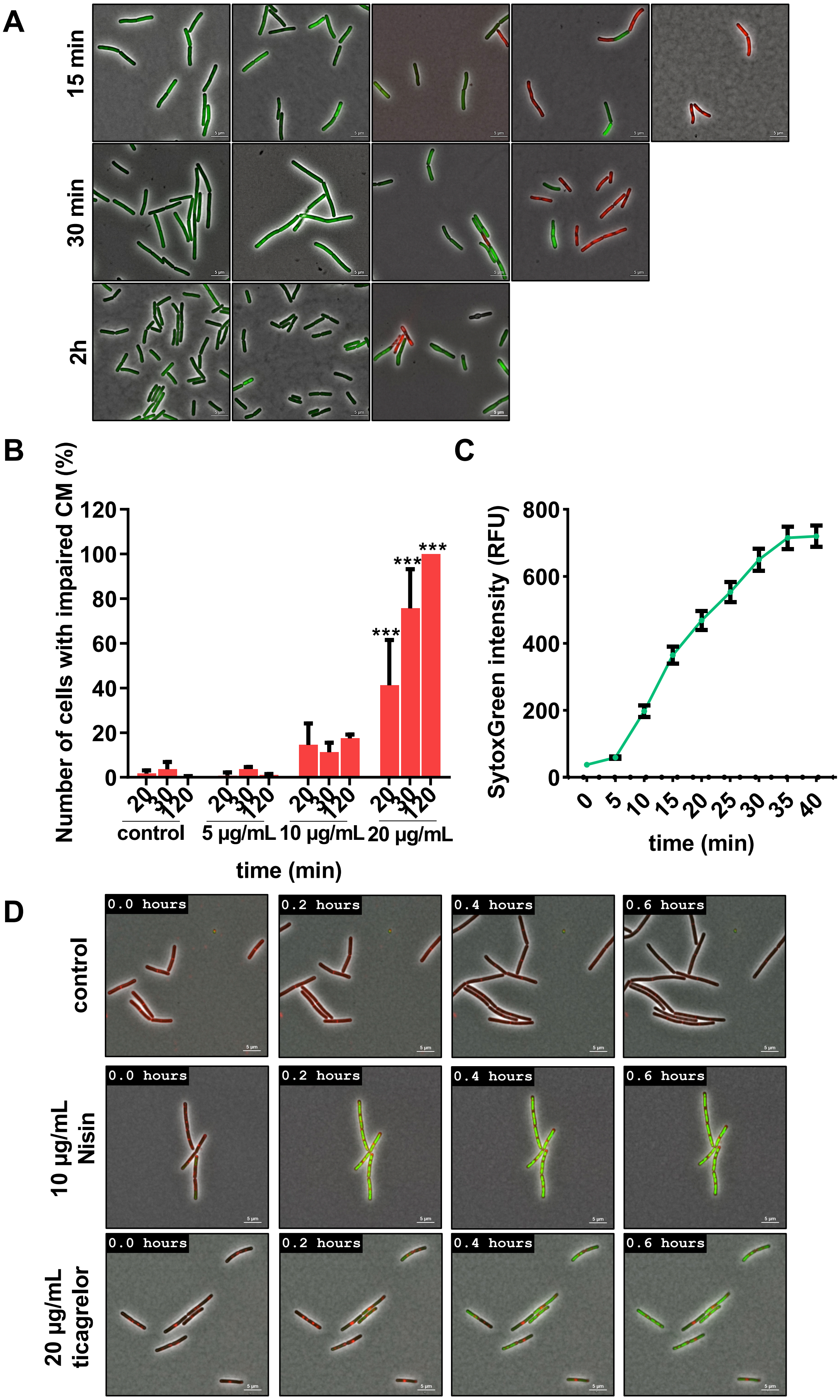
A
15 min
30 min
2h
B
Number of cells with impaired CM (%)
***
control
5 μg/mL
10 μg/mL
20 μg/mL
time (min)
C
SytoxGreen intensity (RFU)
time (min)
D
0.0 hours
0.2 hours
0.4 hours
0.6 hours
control
10 μg/mL Nisin
20 μg/mL ticagrelor

Fig.S4: Effects of ticagrelor on the integrity of the cytoplasmic membrane of *B. subtilis.* Cytoplasmic membrane permeability assayed by propidium iodide (PI, red, membrane-impermeable) and SYTO9 (green, membrane-permeable). (A, B) 15, 30 and 120 min of treatment with ticagrelor with sub-MIC concentrations of ticagrelor compared to the pore former nisin, used as positive control. More than 100 cells were analyzed from 3 independent experiments. Graphs represent mean ± SD. (C, D) Time course of entry of SYTOX Green into the cytoplasm (green, membrane-impermeable dye) for the untreated control (1% DMSO), nisin treatment (10 µg/mL, positive control) and exposure to 20 µg/mL of ticagrelor. Compare Movies S1, S2 and S3 in the Supporting Material for time-lapse imaging. For quantification of the Sytox Green uptake > 200 individual cells were analyzed. Mean ± SEM is presented. Scale bar, 5 µm.

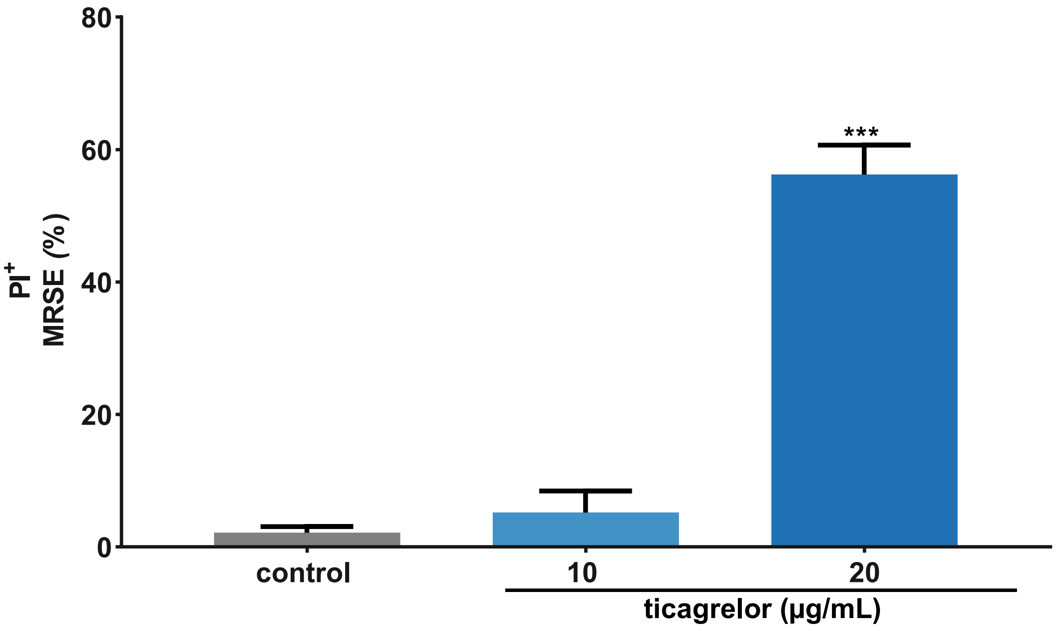

Fig.S5: Quantification of PI positive MRSE after treatment with 10 µg/mL or 20 µg/mL of ticagrelor (N=4). Graphs show mean ± SD, with control (1% DMSO). P-values were obtained via ANOVA with Dunnett's multiple comparison compared to control with ***p-value < 0.001. Propidium iodide (PI).

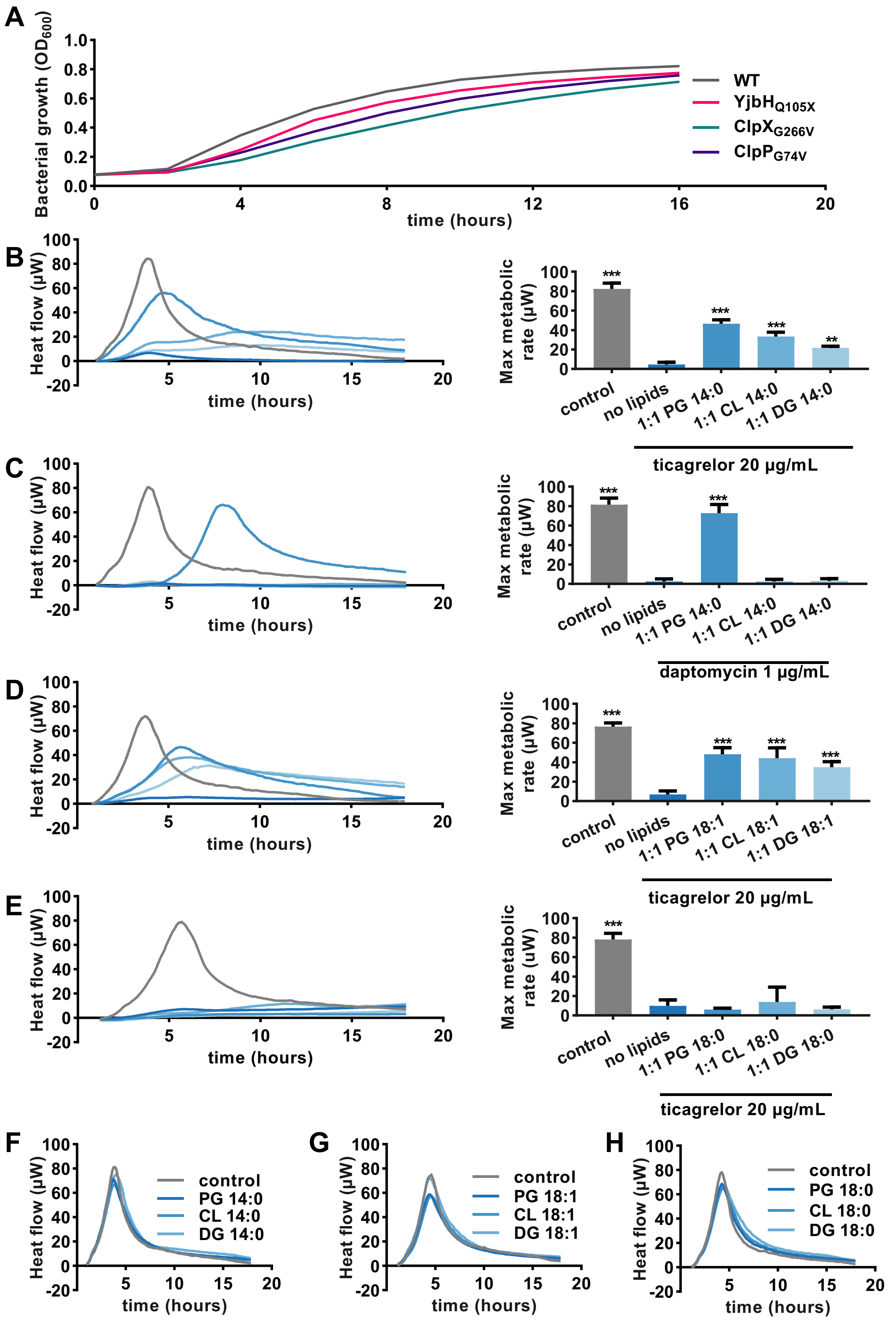
A
Bacterial growth (OD_{600})
time (hours)
WT
$YjbH_{Q105X}$
$ClpX_{G266V}$
$ClpP_{G74V}$
B
Heat flow (μW)
time (hours)
Max metabolic rate (μW)
control
no lipids
1:1 PG 14:0
1:1 CL 14:0
1:1 DG 14:0
ticagrelor 20 μg/mL
C
Heat flow (μW)
time (hours)
Max metabolic rate (μW)
control
no lipids
1:1 PG 14:0
1:1 CL 14:0
1:1 DG 14:0
daptomycin 1 μg/mL
D
Heat flow (μW)
time (hours)
Max metabolic rate (μW)
control
no lipids
1:1 PG 18:1
1:1 CL 18:1
1:1 DG 18:1
ticagrelor 20 μg/mL
E
Heat flow (μW)
time (hours)
Max metabolic rate (μW)
control
no lipids
1:1 PG 18:0
1:1 CL 18:0
1:1 DG 18:0
ticagrelor 20 μg/mL
F
Heat flow (μW)
time (hours)
control
PG 14:0
CL 14:0
DG 14:0
G
Heat flow (μW)
time (hours)
control
PG 18:1
CL 18:1
DG 18:1
H
Heat flow (μW)
time (hours)
control
PG 18:0
CL 18:0
DG 18:0

Fig.S6: (A) Growth curves of WT BAA-1556 and its ticagrelor-resistant clones $Yjbh_{Q105X}$, $ClpX_{G266V}$, and $ClpP_{G74V}$ in the presence of vehicle (1% DMSO). (B) Metabolic activity of MRSA (BAA-1556) in the presence of a 1:1 drug:lipid molar ratio of PG, CL, or DG and ticagrelor or daptomycin, with quantification of maximal metabolic rate (μW) of 14:0 lipids with (B) 20 μg/mL ticagrelor (N=3) or with (C) 1 μg/mL daptomycin (N=3). Metabolic activity and quantification of maximum metabolic activity (μW) of (D) 18:1 lipids (N=6) or (E) 18:0 lipids with 20 μg/mL ticagrelor (N=3). (F-G) Metabolic activity of BAA-1556 in the presence of lipids only including 14:0, 18:1, or 18:0 PG, CL, or DG. Graphs represent bacterial metabolic activity expressed as heat flow (μW) or mean ± SD of max metabolic rate (μW), with control (1% DMSO). P-values were obtained via ANOVA with Dunnett's multiple comparison compared to no lipids, with **<0.01, and ***p-value < 0.001. Phosphatidylglycerol (PG), diacylglycerol (DG), cardiolipin (CL).

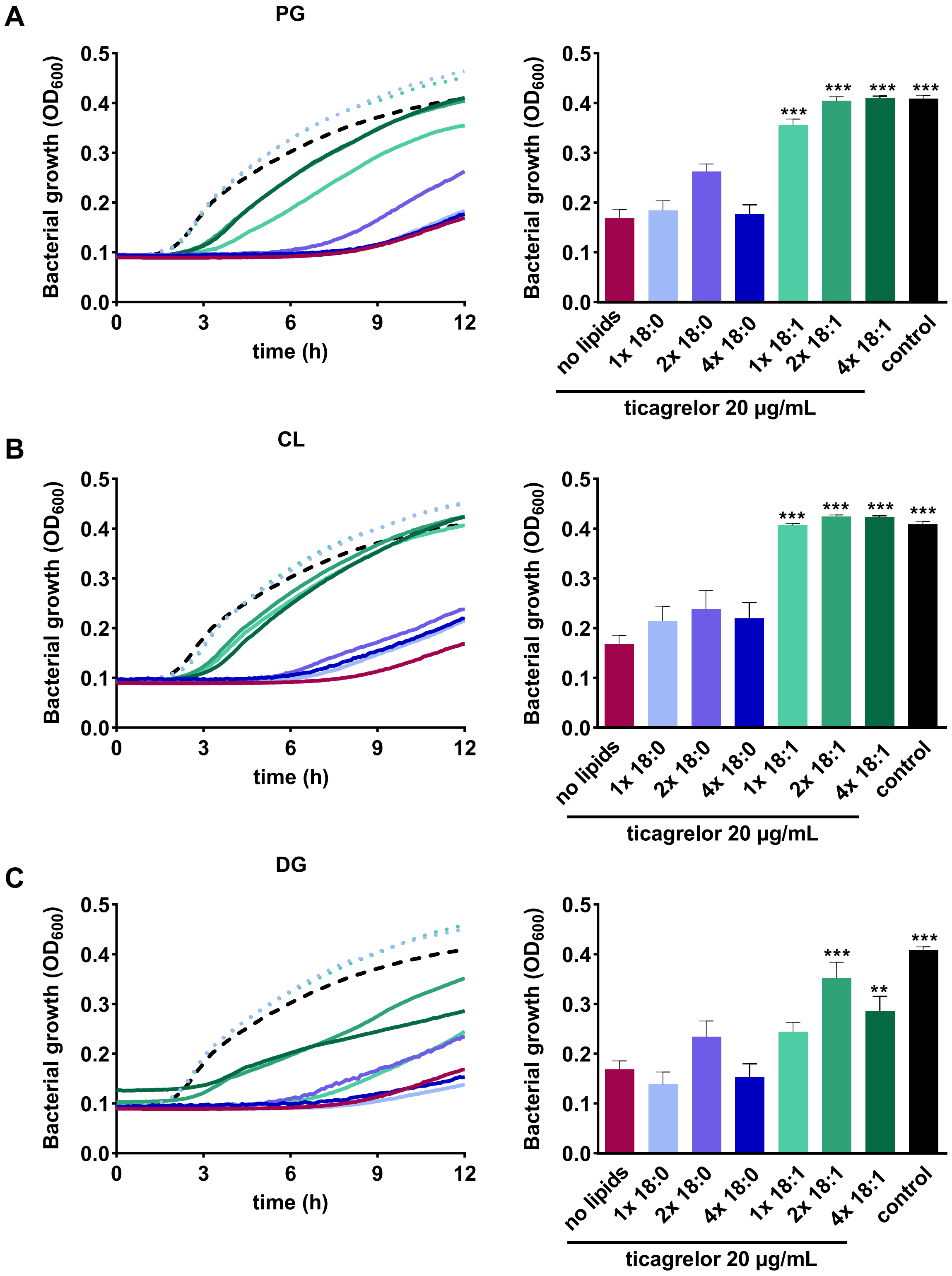
A
PG
B
CL
C
DG
Bacterial growth (OD_{600})
time (h)
0.0
0.1
0.2
0.3
0.4
0.5
0
3
6
9
12
no lipids
1x 18:0
2x 18:0
4x 18:0
1x 18:1
2x 18:1
4x 18:1
control
ticagrelor 20 µg/mL
***
**

Fig.S7. Antagonization of the antibacterial activity of ticagrelor on *B. subtilis* by lipids. Growth (recorded as OD_{600}) of *B. subtilis* 1S34 *liaI-luc* in the presence of 20 µg/mL ticagrelor and the lipids CL (**A**), PG (**B**) or DG (**C**) with either 18:0 (purple) or 18:1 (green) fatty acyl chains. Black dashed lines show untreated growth controls. Dotted lines represent cells treated with lipids only (no ticagrelor). Red lines show cells in the presence of 20 µg/mL ticagrelor (no lipids). Lipids were added in molar ratios of ticagrelor:lipid 1:1, 1:2 and 1:4. Three biological replicates were analyzed (different cultures, different days) with two technical replicates each. Upper row, mean of all growth curves (six per condition). Lower row, endpoint after 12 h of ticagrelor exposure (mean ± SEM). (**A**) Impact of cardiolipin (CL). 18:1 CL fully antagonized ticagrelor activity at 1:1. (**B**) Impact of phosphatidylglycerol (PG). 18:1 PG showed full antagonization at twofold molar surplus. (**C**) Impact of diacylglycerol (DG). 18:1 DG improved growth but could not fully restore it in *B. subtilis*. None of the lipids bearing 18:0 acyl chains yielded significant concentration-dependent effects. P-values were obtained via ANOVA using Dunnett‘s multiple comparison test compared to treatment without lipids with *** $p<0.001$.

Supplemental tables

Table S1: Lipidomic data of MRSA WT and ticagrelor-resistant clones $YjbH_{Q105X}$, $ClpP_{G74V}$, and $ClpX_{G266V}$

See Table S1 excel file.

Table S2: Bacterial strains used in this study

Bacterial strains	Reference
Bacillus subtilis 168	(4)
Bacillus subtilis 1S34 lacZ strains	(1)
Bacillus subtilis 1S34 luc strains	(5)
Bacillus subtilis 1981 (expressing GFP-MinD)	(3)
Staphylococcus aureus subsp. aureus Rosenbach BAA (MRSA)	ATCC-1556
Staphylococcus aureus Mu50 (VISA)	ATCC 700699
Staphylococcus aureus USA300 JE2	(6)
Staphylococcus aureus (MSSA)	NCTC 8325
Staphylococcus aureus BAA-1556-$YjbH_{G105X}$	In house-generated
Staphylococcus aureus BAA-1556-$ClpP_{G74V}$	In house-generated
Staphylococcus aureus BAA-1556-$ClpX_{G266V}$	In house-generated
Staphylococcus aureus (SA113)	ATCC 35556
Staphylococcus aureus 113-ΔMprF	(7)
Staphylococcus aureus 113-$MprF_{T345A}$	(7)
Staphylococcus aureus 113-Δ$MprF_{V351E}$	(7)
Staphylococcus aureus 616	(8)
Staphylococcus aureus-ΔMprF-701	(8)
Staphylococcus aureus-ΔMprF-703	(8)
Staphylococcus aureus (MSSA)	ATCC 6538

Staphylococcus epidermidis (MRSE)	RP62A
Escherichia coli	ATCC 8739

Supplemental movies

Legends

Movie S1: Time course (0 to 0.6 hours) of uptake of SYTOX Green (green, membrane-impermeant dye) into *B. subtilis* in the presence of vehicle (1% DMSO). FM 4-64 (red) stains the membrane to illustrate cell shape. Scale bar = 5μm.

Movie S2: Time course (0 to 0.6 hours) of uptake of SYTOX Green (green, membrane-impermeant dye) into *B. subtilis* in the presence of 20 μg/mL of ticagrelor. FM 4-64 (red) stains the membrane to illustrate cell shape. Scale bar = 5μm.

Movie S3: Time course (0 to 0.6 hours) of uptake of SYTOX Green (green, membrane-impermeant dye) into *B. subtilis* in the presence of 10 μg/mL nisin. FM 4-64 (red) stains the membrane to illustrate cell shape. Scale bar = 5μm.

References

1. Wex KW, Saur JS, Handel F, Ortlieb N, Mokeev V, Kulik A, Niedermeyer THJ, Mast Y, Grond S, Berscheid A, Brötz-Oesterhelt H. 2021. Bioreporters for direct mode of action-informed screening of antibiotic producer strains. Cell Chem Biol 28:1242-1252.e4.
2. Zhang L, Esquembre LA, Xia S-N, Oesterhelt F, Hughes CC, Brötz-Oesterhelt H, Teufel R. 2022. Antibacterial synnepyrroles from human-associated nocardiopsis sp. show protonophore activity and disrupt the bacterial cytoplasmic membrane. ACS Chem Biol 17:2836–2848.
3. Strahl H, Hamoen LW. 2010. Membrane potential is important for bacterial cell division. Proc Natl Acad Sci U S A 107:12281–6.
4. Bohorquez LC, Surdova K, Jonker MJ, Hamoen LW. 2018. The conserved DNA binding protein WhiA influences chromosome segregation in *Bacillus subtilis*. J Bacteriol 200.
5. Urban A, Eckermann S, Fast B, Metzger S, Gehling M, Ziegelbauer K, Rübsamen-Waigmann H, Freiberg C. 2007. Novel whole-cell antibiotic biosensors for compound discovery. Appl Environ Microbiol 73:6436–6443.
6. Fey PD, Endres JL, Yajjala VK, Widhelm TJ, Boissy RJ, Bose JL, Bayles KW. 2013. A genetic resource for rapid and comprehensive phenotype screening of nonessential *Staphylococcus aureus* genes. mBio 4.
7. Ernst CM, Slavetinsky CJ, Kuhn S, Hauser JN, Nega M, Mishra NN, Gekeler C, Bayer AS, Peschel A. 2018. Gain-of-function mutations in the phospholipid flippase MprF confer specific daptomycin resistance. mBio 9:1–12.
8. Jones T, Yeaman MR, Sakoulas G, Yang S-J, Proctor RA, Sahl H-G, Schrenzel J, Xiong YQ, Bayer AS. 2008. Failures in clinical treatment of *Staphylococcus*

aureus infection with daptomycin are associated with alterations in surface charge, membrane phospholipid asymmetry, and drug binding. Antimicrob Agents Chemother 52:269–278.
